# The emergence of highly resistant and hypervirulent *Klebsiella pneumoniae* CC14 clone in a tertiary hospital over 8 years

**DOI:** 10.1186/s13073-024-01332-5

**Published:** 2024-04-18

**Authors:** Sharif Hala, Mohammed Malaikah, Jiayi Huang, Wesam Bahitham, Omniya Fallatah, Samer Zakri, Chakkiath Paul Antony, Mohammed Alshehri, Raeece Naeem Ghazzali, Fathia Ben-Rached, Abdullah Alsahafi, Asim Alsaedi, Ghadeer AlAhmadi, Mai Kaaki, Meshari Alazmi, Baraa AlhajHussein, Muhammad Yaseen, Hosam M. Zowawi, Majed F. Alghoribi, Abdulhakeem O. Althaqafi, Abdulfattah Al-Amri, Danesh Moradigaravand, Arnab Pain

**Affiliations:** 1 Pathogen Genomics Laboratory, Biological and Environmental Sciences and Engineering, King Abdullah University of Science and Technology, 23955-6900 Jeddah, Makkah Saudi Arabia; 2 King Saud Bin Abdulaziz University for Health Sciences, Riyadh, Saudi Arabia; 3 Infectious Disease Research Department, King Abdullah International Medical Research Centre, Jeddah, Saudi Arabia; 4 Ministry of National Guard Health Affairs, Riyadh, Western Region Saudi Arabia; 5 Laboratory of Infectious Disease Epidemiology, Biological and Environmental Science and Engineering (BESE) Division, King Abdullah University of Science and Technology (KAUST), Thuwal, Saudi Arabia; 6 KAUST Computational Bioscience Research Center (CBRC), King Abdullah University of Science and Technology (KAUST), Thuwal, Saudi Arabia; 7 King Faisal Specialist Hospital and Research Centre, Jeddah, Saudi Arabia; 8 College of Computer Science and Engineering, University of Hail, Hail, Saudi Arabia; 9 The University of Queensland, UQ Centre for Clinical Research, Herston, QLD Australia; 10 International Institute for Zoonosis Control, Hokkaido University, Sapporo, 001-0020 Japan

**Keywords:** *Klebsiella pneumoniae*, Precision epidemiology, Whole-genome sequencing, Antimicrobial resistance

## Abstract

**Background:**

*Klebsiella pneumoniae* is a major bacterial and opportunistic human pathogen, increasingly recognized as a healthcare burden globally. The convergence of resistance and virulence in *K. pneumoniae* strains has led to the formation of hypervirulent and multidrug-resistant strains with dual risk, limiting treatment options. *K. pneumoniae* clones are known to emerge locally and spread globally. Therefore, an understanding of the dynamics and evolution of the emerging strains in hospitals is warranted to prevent future outbreaks.

**Methods:**

In this study, we conducted an in-depth genomic analysis on a large-scale collection of 328 multidrug-resistant (MDR) *K. pneumoniae* strains recovered from 239 patients from a single major hospital in the western coastal city of Jeddah in Saudi Arabia from 2014 through 2022. We employed a broad range of phylogenetic and phylodynamic methods to understand the evolution of the predominant clones on epidemiological time scales, virulence and resistance determinants, and their dynamics. We also integrated the genomic data with detailed electronic health record (EHR) data for the patients to understand the clinical implications of the resistance and virulence of different strains.

**Results:**

We discovered a diverse population underlying the infections, with most strains belonging to Clonal Complex 14 (CC14) exhibiting dominance. Specifically, we observed the emergence and continuous expansion of strains belonging to the dominant ST2096 in the CC14 clade across hospital wards in recent years. These strains acquired resistance mutations against colistin and extended spectrum β-lactamase (ESBL) and carbapenemase genes, namely *bla*_OXA-48_ and *bla*_OXA-232_, located on three distinct plasmids, on epidemiological time scales. Strains of ST2096 exhibited a high virulence level with the presence of the siderophore aerobactin (*iuc*) locus situated on the same mosaic plasmid as the ESBL gene. Integration of ST2096 with EHR data confirmed the significant link between colonization by ST2096 and the diagnosis of sepsis and elevated in-hospital mortality (*p-*value < 0.05).

**Conclusions:**

Overall, these results demonstrate the clinical significance of ST2096 clones and illustrate the rapid evolution of an emerging hypervirulent and MDR *K. pneumoniae* in a clinical setting.

**Supplementary Information:**

The online version contains supplementary material available at 10.1186/s13073-024-01332-5.

## Background

The Gram-negative bacterium *Klebsiella pneumoniae* is a major opportunistic human pathogen, increasingly recognized as a healthcare burden globally. It accounts for one-third of Gram-negative bacterial infections in clinical settings [1]. It causes a wide range of acute and chronic infections that are challenging to treat due to its capacity to withstand and adapt to stressors, ultimately enabling resistance against multiple antimicrobials [1]. Resistance against commonly used drugs in *K. pneumoniae* strains is well-recognized globally, and this led to the inclusion of the pathogen on the WHO priority pathogen list, for which immediate novel therapeutic and diagnostic solutions are required [2, 3].

*K. pneumoniae* genomes are globally diverse, and multidrug-resistant clones often emerge locally before spreading globally [4–6]. The pathogenicity and antimicrobial resistance in *K. pneumoniae* is determined through genes and variants carried on mobile genetic elements that disseminate rapidly in the populations [5, 7, 8]. Several recent studies showed that the evolution of clinical strains of *K. pneumoniae* occurs on an epidemiological time scale, in which strains have been shown not only to disseminate across hospital wards rapidly but also to transfer their resistant and virulence genes to other *Klebsiella* and Gram-negative bacterial strains [9, 10]. Furthermore, reports are available about the convergence of virulence and resistance through the colocation of the encoding genes on the same mobile genomic context [11, 12], leading to the formation of hypervirulent (HvKp) and multidrug resistant (MDR) *K. pneumoniae* strains, i.e., resistance to three or more antimicrobial classes [13]. Molecular identification of hvKp strains is performed by detecting specific virulence genes, such as *iuc*, *rmpA*, and *rmpA2* [14]. Among these genes, the *iuc* locus of lineage 1 encoding aerobactin is recognized as one of the most specific virulence markers for hvKp, reported to be prevalent globally [14, 15]. Nosocomial infections caused by MDR and hvKp strains represent a significant public health threat. Outbreak strain transmissions were mainly correlated with asymptomatic gastrointestinal colonization of healthcare workers (HCW) or hospital environments propelled by inherited selective advantages [16]. The long-term care wards and intensive care units (ICUs) primarily accommodate vulnerable populations, including immunocompromised, neonatal, and elderly patients, who are in proximity for an extended duration. Consequently, these healthcare settings are more susceptible to the spread of communicable infections, including hvKp and MDR *K. pneumoniae* strains [17]. Continuous ongoing transmissions underlie outbreaks with limited therapeutic options, resulting in a 30–50% global mortality rate [18]. Therefore, an in-depth understanding of the epidemiology in hospital settings on epidemiological timescales is warranted to contain the outbreaks.

In the Middle East and North Africa (MENA) and West and South Asia, MDR *K. pneumoniae* is among the top infectious organisms reported; however, detailed phenotyping and linking to strain genomic, phylogenetic, and phylodynamic data are lacking or hardly discussed [19]. Although rarely described, *K. pneumoniae* has been shown to have the ability to combine multiple virulence factors and antimicrobial resistance genes [20]. Reports of *K. pneumoniae* outbreaks in Saudi Arabia have indicated the presence of a few unique multi-locus sequencing types (MLST) strains, such as ST29 and ST14, as opposed to globally reported high-risk clones, such as ST258 [21]. Recent reports have identified an abundance of the clonal complex CC14 represented by sequence type ST14 and ST2096 in the MENA region [8, 22]. A recent study on carbapenem-resistant strains in the UAE hospitals implicated CC14 strains in hospital-acquired infections across the country [23]. The study, however, lacked any integration with clinical data. Therefore, the effect of ST2096 genomic characteristics on the clinical significance of the strains remained unstudied. In the broader South Asian region, few studies reported the incidence of CC14 strains, including ST2096 and ST14. A study in India of blood samples between 2016 and 2017 identified ST231, ST14, and ST2096 as the most prevalent sequence types associated with colistin resistance [12]. Another study in India indicated the presence of the multidrug-resistant hypervirulent ST2096 strains in the hospital and community in India [24]. ST2096 strains were also reported in other collections from South Asia and showed genomic signatures of the convergence of resistance and virulence for the strains [15]. However, these studies examined a small sample size, and henceforth, the population dynamics of strains remained unstudied.

This study reports an in-depth genomic epidemiology analysis for a large-scale longitudinal collection of MDR *K. pneumoniae* strains in a major hospital in the west of Saudi Arabia over 8 years between 2014 and 2022. We integrated the genomic data with clinical data from the patients’ electronic health record (EHR). We identified a diverse population underlying the infections over time using a broad range of phylogenetic, phylodynamic, and data mining methods. Despite this, few clones belonging to clonal complex CC14 appeared dominant. Most notably, the ST2096 emerged as an MDR and hypervirulent strain, circulating across hospital wards, and showed significant links with elevated mortality. ST2096 exhibited a higher virulence capability and harbored multiple colistin resistance determinants and plasmid-carrying extended-spectrum β-lactamases (ESBLs) and carbapenemase genes, acquired independently across multiple lineages over time. These results provide insights into the dynamics of an emerging hypervirulent and MDR *K. pneumoniae* strain in a hospital setting.

## Methods

### Sampling and collection specification

As part of the hospital surveillance program, 328 *K. pneumoniae* isolates and their epidemiological data were collected from 239 patients between November 2014 and September 2022. Due to COVID-19 pandemic restrictions, no sampling was conducted in 2019. The isolates were obtained from King Abdulaziz Medical City Jeddah (KAMC-J) and the MNGHA hospitals in Jeddah, Saudi Arabia. The medical city houses a tertiary care hospital with over 600 beds and adult ICUs for medical and surgical patients with an 85-bed capacity. The facility accommodates a significant number of adult LT (long-term) patients distributed across 20 wards, each with the option of single, double, and quadruple beds per room. These facilities cater to the healthcare needs of more than 125,000 National Guard soldiers, hospital employees, and their families residing in the Western region of Saudi Arabia.

We isolated strains from patients, who were defined as infected or colonized by an MDR *K. pneumoniae* by the molecular microbiology laboratory in the hospital. A combination of phenotypic and genotypic methods was used to identify MDR *K. pneumoniae* strains. This included antimicrobial susceptibility testing (AST) and polymerase chain reaction (PCR) for identifying *K. pneumoniae* and β-lactamase genes. Differentiation infections from colonization was performed as per the guidelines of the Infectious Diseases Society of America (IDSA), in which an infection was diagnosed based on clinical symptoms, presence of local and systemic inflammation, high drug resistance level, and isolation of the strain from a sterile body site. Isolation of the bacterium from the blood, urine, wound, or sputum is often regarded as an infection [25]. Over the course of 8 years, the prevalence of *K. pneumoniae* infections versus bloodstream infections relatively stable over the study period. The reported *K. pneumoniae*-related infections in the hospital and cases from bloodstream infections are detailed in Table 1.

**Table 1:** Reported *K. pneumoniae* infections and cases from bloodstream infections over the course of the study

Year	Number of *K. pneumoniae* infections	Number of Cases from bloodstream infections
2014	2123	40
2015	2345	20
2016	2211	30
2017	2398	39
2018	2266	30
2019	2057	40
2020	2444	25
2021	2100	16
2022	2299	60

We collected multiple epidemiological data from patients with clinically diagnosed MDR *K. pneumoniae* including age, sex, length of hospital stays, date of admission, date of discharge, date of sample collection, ward of admission, sampling date, patient status (treatment outcome), and sample type based on their body collection site. Samples were classified into three classes based on their body sites, i.e., blood culture (BC) *n* = 124, urine culture (UC) *n* = 59, and other sites, e.g., from wounds and sputum, *n* = 140 (five samples had no information). We also included the diagnostic notes associated with each patient during hospitalization. Patient status includes the discharged or deceased status by the hospital treatment’s end. The cause of death may or may not be *K. pneumoniae* infections. Community-acquired infections were identified if the infection was detected within 48 h of the patient’s admission to the hospital. The metadata for the samples is provided in Supplemental Table S1.

### Drug susceptibility testing

Samples were isolated from patients from various specimen types after cultures on MacConkey agar (Saudi-prepared media laboratory SPML, Saudi Arabia). The isolates were identified using the automated mass spectrometry microbial identification system VITEK MS from bioMérieux, France. For further identification of individual isolates, conserved 16S rRNA gene(s) were subjected to Sanger sequencing using Applied Biosystems. Antimicrobial susceptibility testing (AST) was conducted using the VITEK2 rapid identification system from bioMérieux, France.

### Short-read sequencing

DNA was extracted using single-colony boiling and GenElute Bacterial Genomic DNA Kit (Sigma-Aldrich, Germany). We sheared the DNA by sonication to reach 300–500 bp product size (Covaris, USA). Manual and automated libraries were prepared using NEBNext Ultra II DNA Library Prep Kit for Illumina (NEB, UK) using BioMek FXP liquid handling automation (Becman Corporation, USA). Samples were sequenced in multiple pools using HiSeq4000 (Illumina, USA) to produce 150 bp paired-end reads.

Reads were controlled for the quality with fastqc package in R (v0.1.3). We then performed de novo assembly using Unicycler (v0.5.0) (https://github.com/rrwick/Unicycler#install-from-source) with default parameters [26]. Assemblies were processed and contigs shorter than 200 bps were excluded from the analysis with Unicycler. We characterized the strains with Kleborate (v2.3.2) to thoroughly identify the sequence and capsule types and the resistome and virolome of the collection [27]. We found 7 unknown K-locus types, and 12 samples showed no detected *wzi* type, which was due to fragmented assemblies (Supplemental Table S1). We used Kleborate to compute virulence and resistance scores, which reflect the presence of relevant AMR and hypervirulent loci. Virulence scores, ranging from 0 to 5, are determined by the presence of specific loci associated with increasing risk, prioritized as yersiniabactin (*ybt*) < colibactin (*clb*) < aerobactin (*i*uc). The virulence scores were defined based on the presence or absence of these loci, as follows: 0 = no yersinabactin, colibactin or aerobactin; 1 = yersiniabactin only; 2 = yersiniabactin and colibactin (or colibactin only); 3 = aerobactin without yersiniabactin or colibactin; 4 = aerobactin with yersiniabactin (no colibactin); and 5 = yersiniabactin, colibactin, and aerobactin [27]. Similarly, resistance scores, ranging from 0 to 3, are assigned based on the identification of genotypes that necessitate the antimicrobial therapy, following the hierarchy of ESBL < carbapenemase < carbapenemase plus colistin resistance. The resistance scores were defined based on the presence or absence of these loci, as follows: 1= ESBL; 2 = Carbapenemase; 3 = Carbapenemase plus colistin resistance; and 0 in other cases [27]. Virulence factors and resistance genes were also identified with srst2 v0.2.0 (www.github.com/katholt/srst2) [28] on databases of Plasmidfinder (v2.1.1) [29], VFDB (v6.0) [30], ResFinder (v4.1) [31] and CARD (v3.2.8) [32]. We used the identity threshold of 90% to identify resistance genes. Furthermore, we annotated the de novo assemblies with Prokka (v1.14.5) [33]. The annotated assemblies were fed into Panaroo to reconstruct the pangenome of strains [34]. The Phytools package (v1.9.25) in R was used to reconstruct the likelihood of ancestral states for the presence of carbapenemase genes [35].

To construct the phylogenetic tree for the collection under study, we first mapped the short-reads against the genome of the reference strain of *K. pneumoniae* Ecl8, a ST375/K2 hypervirulent clone of epidemiological significance causing severe community-acquired infections [36] (accession number: PRJEB401) [37] with the Snippy pipeline (https://github.com/tseemann/snippy) with default parameters. We then computed the pair-wise SNP distance between the strains and reconstructed the neighbor-joining phylogenetic tree using the ape (v5.7.1) package in R [38]. We used results from Kleborate and all nonsynonymous and frameshift, stop-gain, and stop-loss mutations in the *mgr* gene identified by the variant calling pipeline to curate the list of colistin resistance mutations.

We integrated the genetic profile of resistance determinants from the Kleborate with the phenotype data by using the odds ratio function from the Epitools package (v0.5.10.1) in R. Furthermore, we also checked for the significance of associations after accounting for population structure using Scoary (1.6.16) [39] on the Panaroo output and the SNPs identified after mapping the reads to the reference genome. We therefore considered specifically the pairwise *p*-values (worst and best *p*-values as per the tool's definition) which account for the confounding effect of population structure (lineage effect) (www.github.com/AdmiralenOla/Scoary) on associations. We used the ggtree package (v3.8.2) [40] in R to visualize the tree and the associated metadata.

### Transmission analysis and phylodynamic analysis

We conducted phylodynamic analysis on the four dominant STs, i.e., STs with more than ten genomes in the collection of ST14, ST101, ST11, and ST2096 to determine key epidemiological features of the clone and dated phylodynamic tree. We excluded strains within each ST group that did not have a collection date. As we were focused on a single clone, we used a local reference genome to identify variants at a high resolution. We therefore identified the strain with the best assembly statistics, i.e., highest N50, for each clone. We then joined the contigs of the assembly to create a local reference genome. After mapping the short reads for each strain in the clone to this reference genome, we obtained a core genome SNP alignment. We then subject genome alignments to Gubbins (v.3.3.1) [41] with five iterations to remove hypervariable regions. The SNP counts for the major clones were ST101 (669 sites, 55.75 SNP per strain), ST11 (533 sites, 41 SNP per strain), ST2096 (1207 sites, 10.68 SNP per strain), and ST14 (1792 sites, 42.66 per strain). We employed hierarchical clustering method BAPS [42] with one round of iteration using the implementation in R [43] to identify the clusters within the ST2096 clone. Bactdating [44] (www. github.com/xavierdidelot/BactDating) with 10^7^ iterations was used to obtain phylodynamic trees for each clone, from which key epidemiological parameters, i.e., clock rate, effective population size, and most recent common ancestor (MRCA) age were inferred.

We examined the output trees for the significance of the temporal signal by using the roottotip function and checking if the *R*^2^ is more significant than 0. We also compared the model with a tree with no temporal sign using the modelcompare process. We confirmed the convergence of Markov chain Monte Carlo (MCMC) chains by ensuring that the effective sample size (ESS) is more significant than 150 for critical parameters. We used the effectiveSize function on the MCMC output after discarding 10% of the output as a burn-in phase. To dissect the adequate population size changes over time, we sampled population sizes with a non-parametric growth model on the dated phylogenetic tree for the ST2096 clone with the skygrowth.mcmc function and visualized the results with the plot function in the skygrowth package (v0.3.1) [45].

Transmission trees were then reconstructed using the phylodynamic structured coalescent transmission tree inference approach (SCOTTI) implemented as part of BEAST (v2) package [46]. To this end, the output of Gubbins was fed as input in SCOTTI to reconstruct the transmission network for each clone. Patients’ admission and discharge dates were taken as the start and end of the period where patients were exposed to one another and, therefore, the period in which transmissions could occur. SCOTTI employs a statistical framework that models each host as a distinct population and transmissions between hosts as migration events. Samples from the same patients allowed us to model within-host and between-host transmission in a unified framework in SCOTTI. We ran SCOTTI for at least 10^8^ iterations for the four dominant STs and checked the convergence by checking the ESS to be above 150 for critical parameters. We then extracted the network of indirect transmissions, i.e., transmissions through a non-sampled host. After leaving out 10% of the iterations, we combined the trees as a burn-in phase. We aggregated the trees and analyzed and visualized the network using the igraph package (v1.5.1) in R.

### Contextualization of ST2096 samples

We contextualized the ST2096 strains with the available database in the Pathogen Detection database (https://www.ncbi.nlm.nih.gov/pathogens/). We first extracted the epidemiological SNP clusters, including the strains under study in the Pathogen Detection (www.ncbi.nlm.nih.gov/pathogens) database on 06/05/2023. The SNP clusters included all strains with a pairwise SNP distance of a maximum of 50 SNPs. Clusters were computed by the pipeline in the Pathogen Detection portal (www.ncbi.nlm.nih.gov/pathogens/pathogens_help/#references). The high-throughput automated pipeline employs a combined kmer approach and alignment to define clusters. The cutoff is fixed at 50 SNPs and cannot be customized. We provided the accession numbers and metadata for the global strains in the shared clusters in Supplemental Table S1. We extracted the short reads for the external strains and mapped them to the local reference genome for the ST2096 cluster, as detailed above. We then extracted the variant sites and reconstructed the neighbor-joining tree with the ape package in R [38].

We conducted phylogeographic diffusion analysis in discrete space with BEAST (v2) to estimate the ancestral state of locations for the ST2096 strains from Saudi and global strains in the SNP cluster for ST2096. To achieve this, we identified global ST2096 strains with a reported date of isolation, excluding five divergent genomes. Subsequently, we repeated the phylodynamic analysis with BEAST, incorporating the geographical location (country of isolation) as a characteristic inherent to each taxon. We selected a constant population size model with uniform priors on clock rate and a symmetric substitution model with a uniform prior distribution for the discrete trait substitution model underlying the diffusion of the clone in space. We checked for convergence by considering the effective sample size (ESS) for key parameters as detailed above.

### Plasmidome analysis and long-read sequencing

Plasmid DNA was purified from 4 ml overnight culture on LB medium using QIAprep® Miniprep kit according to the manufacturer protocol. We measured plasmid concentration and purity using Qubit HS dsDNA kits and spectrophotometry at dual UV light (260/280). Sequenced libraries for different plasmid isolates were prepared, multiplexed using 96-plex Rabid Barcoding Kits and loaded into MinION flow cells (Oxford Nanopore Technologies) and ran for 48 h following the manufacture protocol. We then conducted hybrid assembly with Unicycler with the conservative option to retrieve the plasmid assemblies. The assembled contigs were screened for full copy of origin of replications, virulence factor genes, and antimicrobial resistance genes in the plasmids with blast using the abovementioned databases.

The assembled genomes were visualized and checked with Bandage (v0.9.0) [47]. We annotated the assemblies for potential plasmids with Prokka and reconstructed the pan-genome of the plasmids with Panaroo [34]. We then conducted the co-evolution between the genes analysis with Sydrpick algorithm [48]. Cytoscape (v3.10.1) (https://cytoscape.org/) was employed to visualize the network. The Spydrpick algorithm uses pairwise mutual information (MI) values to identify interactions that appear significantly different from the background distribution. Plasmids were visualized with Proksee portal (www.proksee.ca) [49].

### Diagnostic term text analysis

We analyzed the diagnostic notes for each patient to extract relevant diagnostic terms, comorbidities, and symptoms. We extracted the International Classification of Diseases (ICD version 10) codes, i.e., globally used codes for various diseases, conditions, and health-related issues, for each patient from the EHR data.. For 18 patients, no ICD-10 code was available. We then computed the Charlson comorbidity index from ICD-10 codes using the “comorbid_quan_deyo” function in the “icd” package (version 4.0.9.9) (www.jackwasey.github.io/icd/) in R for patients with different ST strains. The Charlson comorbidity index is a widely used medical scoring system that predicts the 10-year mortality for a patient with a range of comorbid conditions, including heart disease, AIDS, cancer, and others.

We also employed a natural language processing approach to extract all medically relevant terms and compare the importance for patients with ST2096 strains and other STs. To this end, we first processed the diagnostic notes with a text processing pipeline using the tidytext package (v0.4.1) in R. The text was processed by removing special characters and stop words, i.e., common insignificant words. We employed three corpus libraries of known biomedical terms of BC5CDR, en_core_sci_lg, and ner_bionlp13cg_md and extracted relevant terms for diseases and symptoms. The BC5CDR (BioCreative V CDR corpus) is a disease name entity recognition, which is a library of 1500 PubMed articles with 4409 annotated chemicals, 5818 diseases, and 3116 chemical-disease interactions [50], while the core_sci_lg and ner_bionlp13cg_md libraries included 600 k generic medical words and genetic terms for 16 types of cancer, respectively [51]. The list of terms associated with each patient is provided in Supplemental Table S1. To determine the importance of the extracted terms with the infection by different STs, we computed the term-weighting schemes of frequency–inverse document frequency (tf-idf) values. To this end, we first created a separate text corpus for ST2096 and other STs, containing all diagnostic notes for the patients with the same ST. The tf–idf values increase with the number of times a word appears in the document, i.e., textual corpus for each ST, and is offset by the number of documents, i.e., number of textual corpuses for each ST, in the corpus that contained the term. Compared with those for the other STs, the feature provides a proxy for the overrepresentation of a particular term for the notes for ST2096.

### Integration of resistome and virulome data with clinical metadata

We evaluated the odds ratio of death associated with colonization by the major STs using the odds ratio function from the Epitools package (v0.5.10.1) in R. To ensure the reliability of our findings and account for potential confounding effects of demographic and clinical features, we further validated the results of the odds ratio calculation with a binary logistic regression analysis. This analysis considered the mortality outcome as a binary response variable. We examined the significance of resistance in combination with potential confounding factors, such as ST, age, gender, body site of infection, and hospital ward:

$$In\;Hospital\;death\sim ST+\text{age}+\text{gender}+body\;site+Charlson\;comorbidity\;index$$

In the above equation, ST, gender, and body site were treated as categorical independent variables and, therefore, factorized. $\mathrm{Charlson comorbidity index}$ is the continuous comorbidity score computed from ICD10 values. We grouped STs with less than ten representative genomes in one group. The regression coefficient *β* associated with the in-hospital death for each ST was extracted*.* This coefficient corresponded to the change in log odds of death when colonized/infected by each ST.

### Statistical significance analysis

Different statistical tests were used to assess the significance of the findings. For the ratio and mean difference significance between groups, we employed a one-sided proportion test and Wilcoxon signed-rank test, using prop.test and wilcox.test in R. For Bayesian analyses, the assessment of significance was conducted based on the 95% highest probability density (HPD) credible intervals. If the HPD intervals for the two groups did not overlap, we reported a significant difference between the two groups. For the term importance analysis from the natural language processing pipeline, we conducted bootstrapping 100 times and computed the confidence intervals from the bootstrapped data for the importance (tf-idf) of each term.

## Results

### Population is diverse with some clones expanding

We sequenced the genomes of 328 samples over eight years from various body sites, including urine culture (UC) (*n* = 59) and blood culture (BC) (*n* = 124) and other sources (*n* = 140) (Supplemental Figure S1A), taken from 239 patients. The collection demonstrated high diversity, comprising 34 different STs. From 54 patients more than one strain was recovered. In 11 patients, strains from more than one ST group were isolated from the patients. Moreover, in 22 patients, strains with the same ST were recovered from different body sites, showing the extent of within-host diversity and cross-infection. Four STs had more than ten isolates in the collection (ST11 *n* = 13, ST101 *n* = 14, ST14 = 45, ST2096 *n* = 120), which altogether constituted 58% of total population. K-locus diversity shows correspondence of dominant K17, K64, K47, and K2 loci at the frequencies of 100% (14/14), 99% (119/120), 77% (10/13), and 98% (44/45), in ST101, ST2096, ST11, and ST14, respectively. The STs’ absolute and relative frequencies (absolute frequency divided by total absolute frequencies) exhibited variations over time (Supplemental Figure S1B). In 2014 and 2015, ST14 was the most prevalent ST, and it was still detectable in 2022. ST2096 strains were detectable in 2015 and constituted most of the collection in 2016, 2017, and 2018 but declined in 2020. ST101 strains were only detected in 2017 and 2018, while ST11 was first detected in 2020. All dominant STs had been previously reported in outbreaks in Europe and Asia [52–55]. We extracted the epidemiological clusters (see “Methods”) from the Pathogen Detection database to reveal the recent links between the collection and the global population. We found five STs, i.e., ST307, ST14, ST985, ST2096, and ST101, to be part of broader epidemiological clusters (SNP clusters) (Supplemental Figure S1C). While few environmental strains were found in the ST307 and ST101 clusters, the other strains originated from clinical settings across countries on different continents, indicating that our collection is part of globally circulating clinical clones (Supplemental Figure S1C).

### Phylogenetic tree structure and epidemiological features of major clones

**Fig. 1:**
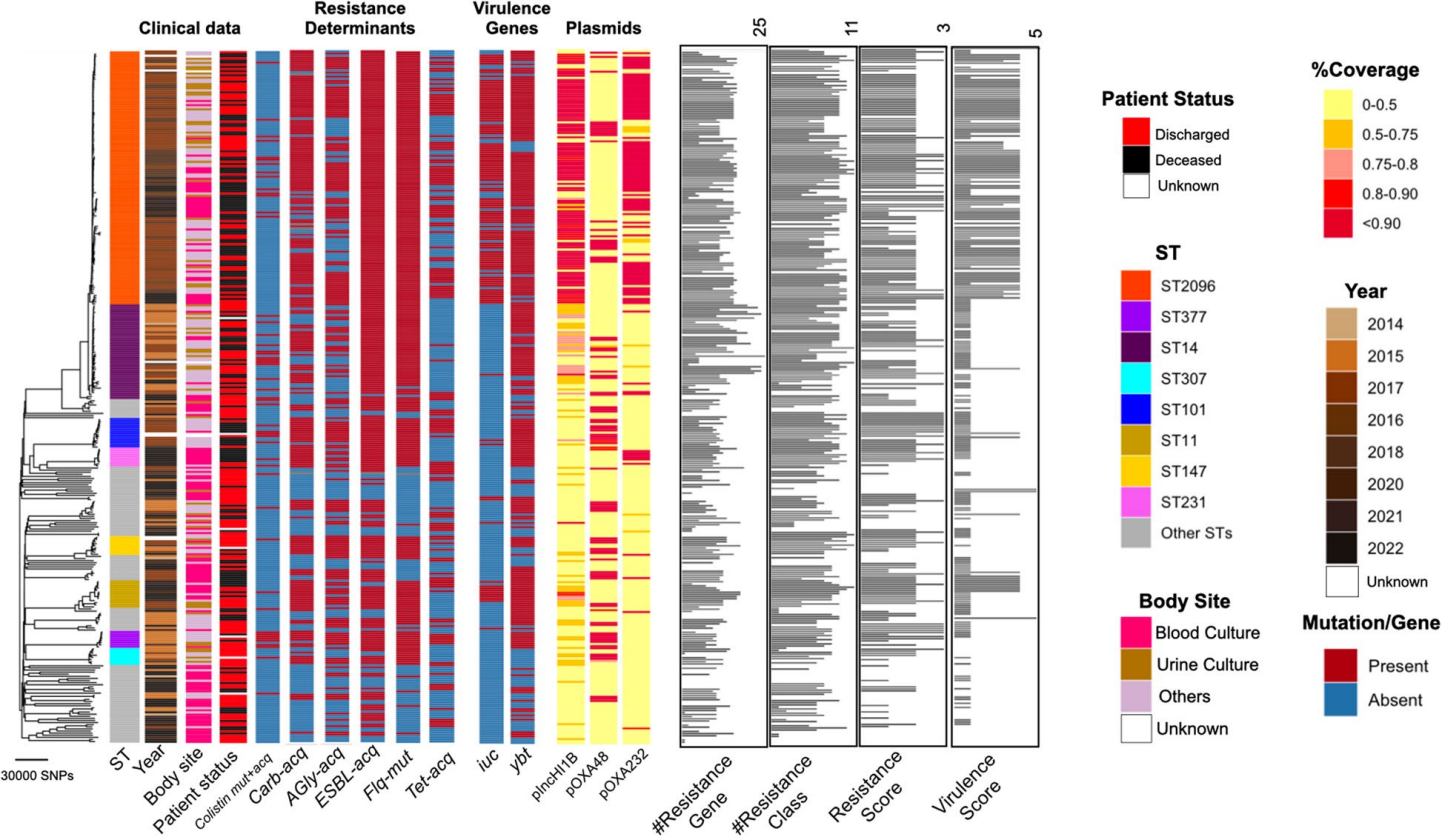
Phylogenetic tree of the collection and the metadata. The neighbor-joining tree for 328 genomes under study. The tree was reconstructed from the pairwise sequence (SNP) distance values, by ape package in R. The SNPs were called with Snippy (see “Methods”). We rooted the tree using midpoint rooting. The resistance profile shows the distribution of major resistance determinants, i.e., Carb (carbapenemase), ESBL, AGly (aminoglycosides), Flq (fluroquinolone), and Tet (tetracyclines) (see Supplemental Table S1 for the full list of resistance determinants). The virulence and resistance scores were computed from Kleborate, based on the presence of key virulence and resistance determinants (see “Methods”) [27]. Numbers on the distributions of virulence score and count of resistance genes and classes and resistance score show the absolute value. The terms “mut” and “acq” stand mutations and acquired resistance genes, respectively. The coverage strips show the percentage of sites of the plasmid sequences to which short reads for each strain were mapped

The phylogenetic tree for the 328 *K. pneumoniae* samples under study implies a diverse population underlying the infections in the hospital, with some lineages undergoing recent clonal expansion (Fig. 1). A total of 282,207 SNP sites were found in the collection after mapping to the reference genome. The major clones of ST2096 (8801 SNPs/73.34 SNPs per isolate/3% of total SNPs), ST14 (10,076 SNPs/223.91 SNPs per isolate/4% of total SNPs), ST11 (10,591 SNPs/814.69 SNPs per isolate/4% of total SNPs), and ST101 (4438 SNPs/317 SNPs per isolate/2% SNP per isolate) constituted major clones. The expanding CC14 clones belonging to STs, i.e., ST14 and ST2096, had a lower SNP count per isolate value and contained a smaller portion of total population variation (*p*-value from proportion test < 0.001), compared to ST11 and ST101 (Fig. 1). The distribution of epidemiological features for major STs implies that ST2096 formed more recently than other ST11 and the other sister CC14 clade of ST14 (Fig. 1, Figure S2A), as the 95% HPD interval were not overlapping for the estimated most recent common ancestor (MRCA) ages for the clones. Despite a comparable population size for ST2096 compared with other major STs (ST14, ST11, and ST101), the ST2096 showed a lower transmission rate than all other STs and lower clock rates than ST14 and ST11 (Figure S2A).

### Resistance against multiple antimicrobial classes is common

Our collection of 328 isolates showed a high level of resistance against different classes of antimicrobials. The phenotypic results revealed that across different STs, 72%, 90%, 87%, 90%, and 58% were resistant to aminoglycosides, β-lactams, trimethoprim, ciprofloxacin, and colistin, respectively (Figure S3A). Among the dominant STs, except for ST11, which is susceptible to colistin and tigecycline, a high resistance level was consistently observed across different STs. The prevalence of ESBL genes among the isolates was 58%, which varied across major STs (ST2096: 65%, ST14: 44%, ST11: 69%, ST101: 84%). The most common ESBL gene was *bla*_CTX-M-15_ (*n* = 186/328, 56%). Carbapenem resistance genes occurred in 62% of the strains, with prevalent STs mostly harboring the genes (ST2096: 0.85, ST14: 0.59, ST11: 1.00, ST101: 0.93). Carbapenem resistance genes differed between the clones: for ST2096 strains, *bla*_OXA-48_ (21/99) and *bla*_OXA-232_ (78/99) were prevalent. *bla*_OXA-48_ was the dominant carbapenemase in ST101 (12/14). In contrast, for ST14, *bla*_NDM_ (21/45) and for ST11 *bla*_KPC-2_ (ST11: 9/13) were most prevalent. The integration of genotype and phenotypes for resistance results show significant odds ratio values greater than 1 (95% confidence interval above one) for 4 groups, 5 groups, and 4 groups of β-lactams for acquired β-lactams (other than ESBLs and carbapenemase), acquired carbapenemases, and ESBL genes, respectively (Figure S4A). The presence of acquired β-lactams (other than ESBLs and carbapenemase), acquired carbapenemases, and ESBL genes explained an average of 76%, 71%, and 74% of resistance phenotypes, respectively (Figure S4A). The presence of osmoporin (Omp) mutations is also linked with an odds ratio significantly above 1 (95% confidence interval above 1) to three β-lactam antimicrobials and is present in on average 74% of resistant strains (Fig. S4B). Among the variants in the *omp* gene, the insertions in the transmembrane β-strand loop in OmpK36 (OmpK36GD) [56] and frameshift mutations in OmpK35 appeared to have significant odds ratio for resistance against five and four drugs and had an average resistance detection rate of 63% and 24%, respectively. These values were higher than those for frameshift mutations in OmpK36, which were not linked to higher resistance for any β-lactam, despite a previous report on the ST258 strain (Figure S4A and B) [57]. The presence of SHV mutations and β-lactamase located on the chromosome are less significant. Fluoroquinolone resistance mutations and aminoglycoside resistance genes were also prevalent in the population at 74% and 84% of the genomes, respectively (Supplemental Figure S3B). The presence of aminoglycoside and fluoroquinolone resistance genes is linked with significantly higher odds ratios for gentamicin and ciprofloxacin, which were present in 76% and 78% of resistant strains, respectively. The odds ratios were weaker for fluoroquinolone resistance-acquired genes, which were found in 27% of the resistant strains (Figure S4B). Colistin resistance mediated by acquired *mcr* genes occurred in five strains, distributed across multiple STs, while colistin mutations, predominantly in the *mgrB* gene, were found in 65/328 (20%) of the population, with 23% (27/120) in ST2096, 24% (11/45) in ST14, 15% (2/13) in ST11, and 78% (11/14) in ST101 [58, 59]. Among the acquired resistance genes, *bla*_OXA-1_ was found to be significantly associated with resistance to cephalosporins and amoxicillin/clavulanate after accounting for lineage effects (*p*-value < 0.05 from GWAS analysis). Because the collection was multidrug-resistant (MDR) to different classes of antimicrobials, the known resistance determinants showed associations with lineages and clones. Overall, the phylogenetic distribution of some of the key resistance genes, e.g., ESBLs and resistance genes/mutations for carbapenemases, aminoglycosides, fluroquinolone, tetracycline, and colistin, demonstrated both cases of the acquisition of the genes/mutations for strains within recent clades and the persistence of the genes in the lineages (Fig. 1).

### Virulence factors are common, with some clones having genetic hallmarks of hvKp

Besides a high resistance level, the collection also included multiple virulence factor genes associated with the hypervirulent phenotype (Figure S3B). Consistent with this, the *iuc* locus was present in the absolute majority of ST2096 (94/120 or 78%) and, to a lesser extent, in ST11 strains (9/13 or 69%) (Supplemental Figure S2B). However, ST101 and ST14 contained only 2/14 (14%) and 1/45 (2%) *iuc*-positive strains, respectively, and the prevalence in the background population was similarly as low as 3%. The *rmpADC* genes were mainly present in ST11 (9/13 or 69%) and 3 strains from other lineages. The yersiniabactin gene (*ybt*) cluster was observed in most strains (72.0%) (Supplemental Figure 3B). The most prevalent yersiniabactin lineages were lineages ybt14 (158), *ybt9* (36), *ybt10* (22), and *ybt13* (10), which were chromosomally embedded in integrative conjugative elements (ICEKp): ICE*Kp5*, ICE*Kp3*, ICE*Kp4*, and ICE*Kp2*, respectively. The frequency of yersiniabactin in major STs was 92%, 90%, 100%, and 100% for ST2096, ST14, ST11, and ST101, respectively, which was much higher than the rest of the population (47%), indicating that these prevalent STs represent high-risk strains. The occurrence of colibactin (*col)* and salmochelin (*iro*) loci was low across the population, at 1% for both loci (3/328) (Figure S3B). The *iro1* locus was not observed in the major STs and only occurred in two isolates of ST23. The same strains carried the colibactin locus of *clb2* for ST23 and *clb3* for ST268. ST23 strains also carried the *iuc* locus, which rendered them highly virulent (virulence score of 5, i.e., the presence of yersiniabactin, colibactin, and aerobactin loci), although they lacked major resistant genes.

### Convergence of virulence and resistance for three common STs

**Fig. 2:**
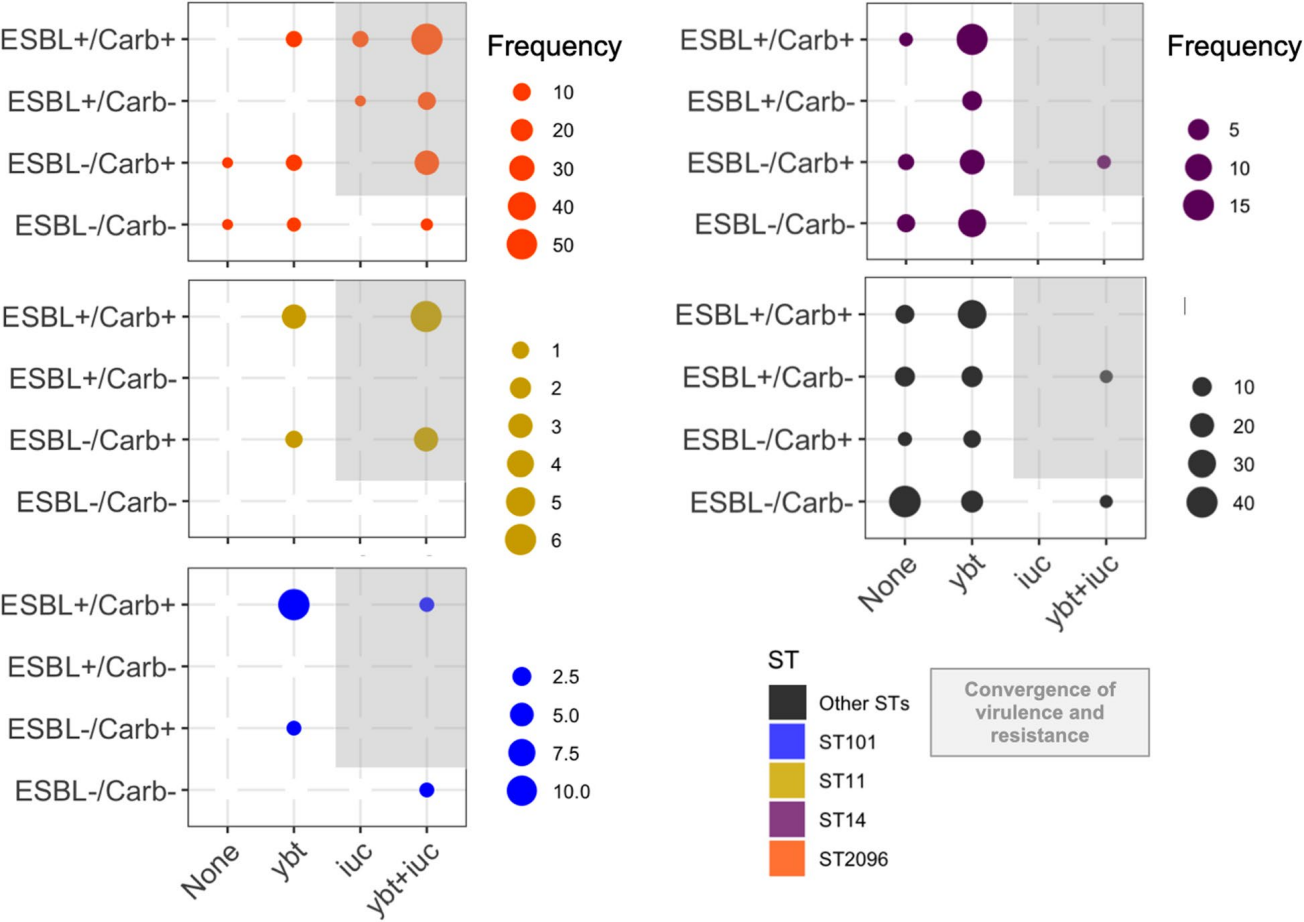
Genotypic convergence of resistance and virulence. The panel corresponds to genomes with and without ESBL or carbapenemase genes. The bubbles correspond to the distribution of genome harboring virulence genes for the siderophores of aerobactin (*iuc*) and yersiniabactin (*ybt*). The genes were identified using Kleborate (see “Methods”). The shaded region shows the strains with convergence of virulence and resistance as per the score computed by Kleborate, based on the virulence and resistance gene profiles

The prevalence of hallmark genes for virulence factors and resistance genes prompted us to dissect the co-occurrence of these elements in the same genome, resulting in strains posing a particular public health threat [15]. The *ybt* virulence locus was observed in ESBL + strains of major STs (ST2096 *n* = 58/76, ST14 *n* = 19/20, ST101 *n* = 11/12, ST11 *n* = 3/9) and in carbapenemase-producing (CP) strains of three STs (ST2096 *n* = 3/99, ST14 *n* = 15/25, ST101 *n* = 4/13) (Fig. 2). The prevalence of *iuc* locus was less common in ESBL strains and was restricted to three STs (ST2096 *n* = 50/76, ST11 *n* = 4/9, ST101 *n* = 1/9), and in carbapenem-resistant strains, it was found in two STs (ST2096 *n* = 2/99, ST101 *n* = 4/13). Among ST2096, ST14, ST11, and ST101 strains, the convergence of resistance and hypervirulence (organisms carrying *iuc locus* plus ESBL and/or carbapenemase-producing, shaded box in Fig. 2) was found in 95/120 (78%), 1/45 (2%), 9/13 (70%), and 1/14 (7%) cases, respectively. The numbers for ST2096, ST11, and ST101 were much higher than for the other STs, which was 2/148 (1%). The ST2096 clone had a higher virulence and resistance scores, and resistance gene and classes count than other non-major STs (*p*-value Wilcoxon test < 0.001) (Figure S2B). Compared to the major STs, ST2096 has a higher virulence and resistance score and resistance classes than the ST14 clade (*p*-value Wilcoxon test < 0.05 and < 0.001, respectively) (Figure S2B). ST101 had a significantly higher resistance score than ST2096 (*p*-value Wilcoxon test < 0.001). In other features, the metric for virulence and resistance genes were comparable between the clones. The findings highlight the high dual resistance and virulence ability of ST2096.

### Population dynamics of the ST2096 clones indicate independent emergence of resistance and clonal expansion

**Fig. 3:**
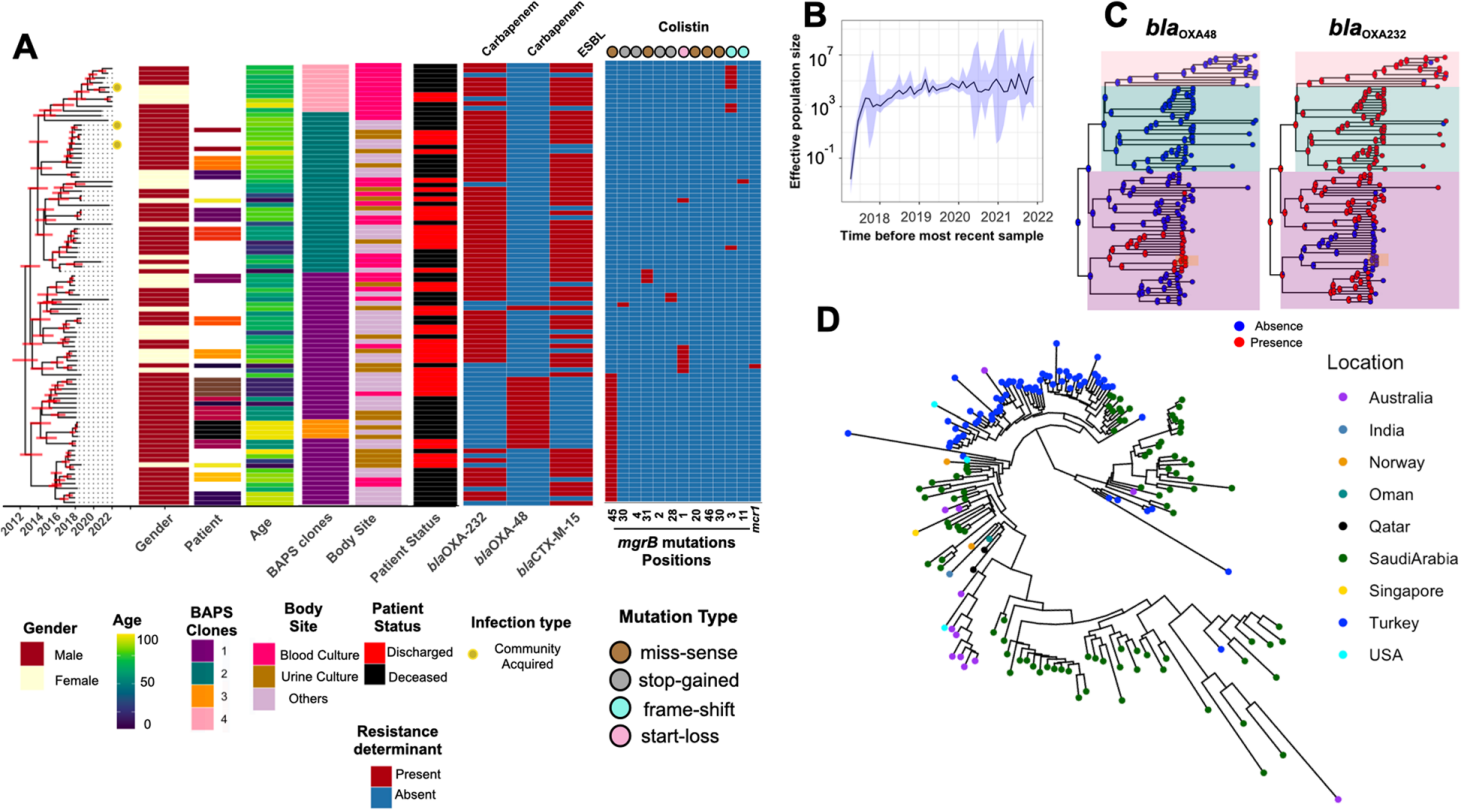
The phylodynamic of ST2096 strains. **A** The Bayesian phylodynamic tree for the ST2096 strains. The horizontal red lines show 95% Highest Posterior Distribution (HPD) for the age of the internal node. We showed the distribution of carbapenemase, ESBL positive, with the acquired genes for aminoglycoside and the distribution of missense, stop gained, and frameshift mutations in the *mgr* gene and the acquired *mcr* gene. Colors on the patient panel show whether the isolates were retrieved from the same patients. We did not show the results for patients with one representative strain. **B** Bayesian skyline plot for the size of the population over time. The shaded region corresponds to 95% confidence interval. **C** Ancestral state reconstruction for the presence of the carbapenemase genes for the ST2096 clone. The pie charts represent the likelihood of the ancestral state. The tree is a Bayesian tree in (**A**). The tree tips show the presence/absence status of the genes. The clade colors show the BAPS clusters in (**A**). **D** Contextualization of the ST2096 clone with the global population. The neighbor-joining tree was reconstructed from the SNP distance matrix for the ST2096 cluster. Five divergent strains were removed before reconstructing the tree

Given the significance of the ST2096 clone, our focus for the rest of the study was on this clone. We further examined the population dynamics of the clone that has expanded since 2012 in the hospital (Fig. 3A). The adequate size of the population indicated a rapid initial expansion, followed by a stable population size over time (Fig. 3B). Samples from body sites and patients age groups were distributed across the phylodynamic tree reconstructed from 1207 SNP sites, corresponding to 10.68 SNPs per strain. The variation was also observed within the subclones of the ST2096 clone. We identified four clusters using the BAPS method (Fig. 3C). Clusters 3 (*n* = 4) and 1 (*n* = 45) included a total of 49 strains. Clusters 4 and 2, with sizes of 10 and 34, respectively, formed two distinct clades. Although samples belonging to the same patient mostly grouped together on the phylogenetic tree, for 3 out of 16 patients’ samples, lineages for strains from the same patients were mixed with lineages for samples from other patients (Fig. 3A). For three patients, the community routes of infection acquisition were confirmed, i.e., infections were detected within 48 h after the patient’s admission, consistent with previous reports on the occurrence of hvKP in the community [21]. The distribution of resistance genes, along with colistin resistance mutations, suggested high dynamics of gene/mutation acquisition during the relatively short period of 8 years (Fig. 3A). *bla*_OXA-48_ was found in cluster 1/3 while *bla*_OXA-232_ was found in all clusters, except cluster 3. Ancestral state reconstruction of these genes indicated that the *bla*_OXA-232_ gene was lost in some lineages over time, while one expanding lineage acquired the *bla*_OXA-48_ gene (Fig. 3C). Notably, isolates with the *bla*_OXA-232_ gene predominantly harbored the ESBL *bla*_CTX-M-15_ gene. Except for one instance of the gain of the *mcr* gene, colistin resistance primarily occurred through multiple mutations in *mgr*, including four stop-gains, five missenses, two frameshifts, and one start-loss mutation, in independent lineages (Fig. 3A). This pointed to the intense selective pressure for these resistance variants from antimicrobial treatment (Fig. 3A).

### Mixing of ST2096 samples with global samples

The ST2096 clone in the collection under study was part of a SNP cluster (cutoff to define the cluster 50 SNPs, see “Methods”) with samples predominantly isolated from the Middle East and South Asia, including India, and a few cases collected from countries further afield (Fig. 3D). Remarkably, the Saudi strains appeared to be ancestral to a substantial clade of samples from Turkey that were reported in 2022. This observation suggests multiple instances of the strains being introduced inside and outside Saudi Arabia, indicating the rapid dissemination of these strains and highlighting the global clinical significance of ST2096. Moreover, ST2096, with its similar resistance and virulence characteristics, has been recently reported as a cause of hospital outbreaks in Turkey [60] and isolated from both community and clinical settings in India [15, 24]. The phylodynamic of the total global ST2096 isolates shows that on five occasions, as shown in the phylodynamic tree, samples from Saudi Arabia appear to serve as ancestors to those from Turkey (Figure S5). All these findings underscore the importance of ST2096 as an emerging strain of global clinical significance.

### ST2096 spreads across wards with a higher transmission rate between bloodstream infection and deceased patients

**Fig. 4:**
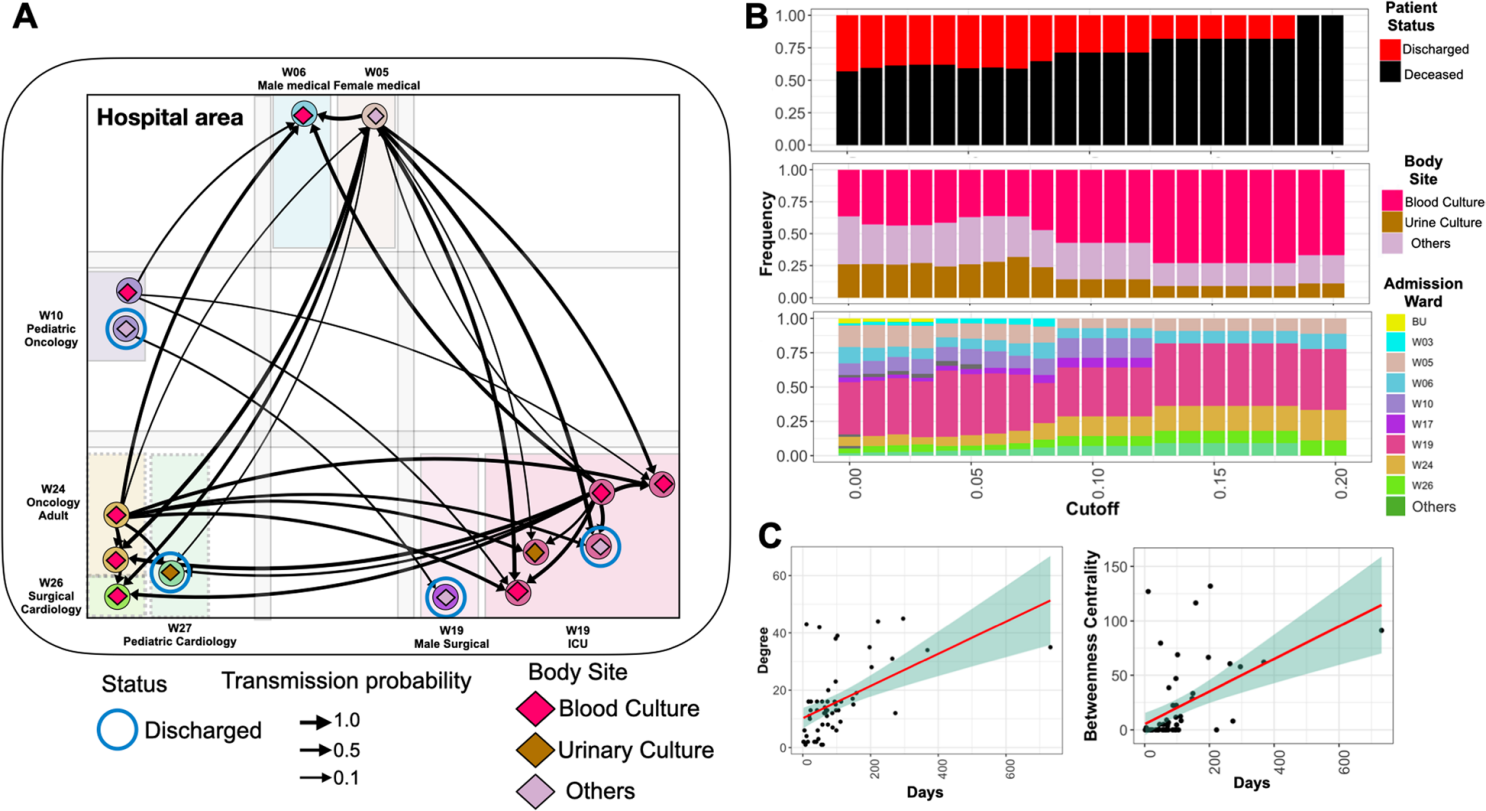
Transmission dynamics of ST2096 clone. **A** Inferred transmission network of ST2096 in the hospital under study (for indirect transmission). Nodes and edges represent patients and indirect transmission routes with a probability more significant than the cutoff of 0.1, respectively. The thickness of the edges corresponds to the probability transmission. Patients who survived until the end of hospital treatment are marked with blue circles. Other patients had a deceased status. **B** The distribution of patient status, body site, and admission hospital ward in networks with different cutoffs for the transmission routes. **C** The relationship between the total node degree and betweenness centrality with hospitalization length, measured in days. The red line shows the fitted linear regression, and the shade shows 95% confidence interval. For a definition of degree and betweenness centrality, refer to “Methods”

To examine the dynamics of the ST2096 clone in the hospital setting, we reconstructed the transmission network from the dated Bayesian transmission tree generated with a structured coalescent inference method (see “Methods”). Figure 4A illustrates the transmissions network in which transmissions with a probability greater than 0.1 across hospital wards are filtered. Out of the 14 patients in this network, the majority (nine) had bloodstream infections, and 4 four survived by the end of the treatment. There was no clustering of links based on the ward of admission; instead, patients admitted to distant wards constituted the transmission routes. This pattern was consistent regardless of the probability cutoff used for the transmission network. Stronger transmission links between patients with ST2096 occurred more frequently between patients who reported a hospital death and those with bloodstream infections (Fig. 4B). The distribution of hospital wards did not appear to influence the transmission probability, indicating that the strains spread freely across wards (Fig. 4B). This pattern was also observed for other major ST clones, which appeared to circulate across different wards (Figure S6). However, in contrast to ST2096, no link was found between a patient’s in-hospital status or site of infection and the probability of transmission for other ST clones, as the distribution of isolates in the network remained the same for the other STs, regardless of different cutoff values for the probability of transmission routes (Figure S6). Our results also indicated a strong link between the length (duration) of hospitalization and the role of the patient as the spreader in the transmission networks for the ST2096 clone (Figure S6). We observed a significant relationship between both the node degree (the number of incoming and outgoing potential transmission links) and betweenness centrality (which corresponds to the degree to which a patient stands between transmission routes) and the number of days of hospitalization (*p*-value for the significance of the slope < 0.01) (Fig. 4C). A high node degree indicates high connectivity, observed for hyper-spreaders, i.e., an individual with the potential to infect a disproportionately large number of people compared to the average infected person, whereas nodes with high centrality are bridges for transmission across the network. The regression analysis indicated that a single day of hospitalization increased the number of connections and betweenness score by an average of 0.05 (standard deviation: 0.01) and 0.15 (standard deviation: 0.03), respectively. Altogether, these results demonstrate a distinctive in-hospital epidemiology pattern for ST2096, compared to other frequent clones, and suggest a higher transmissibility for the ST2096 strains for patients with a more extended hospitalization period.

### Plasmids distribution implies links with previous collection

We examined the entire plasmid content of the ST2096 clone. Among the 69 samples, from which we were able to retrieve plasmid DNA, we detected 3 types of plasmids, including pIncHI1B plasmid in 47 genomes, pOXA48 in 8 genomes, and pOXA232 (pColKP3) in 8 genomes. The pOXA48 and pOXA232 plasmids carried different carbapenemase gene of *bla*_OXA-48_ and *bla*_OXA-232,_ respectively. Notably, despite the difference in the size of the pOXA48 and pOXA232 plasmids, both carried the carbapenem resistance gene (Figure S7A). While pOXA48 was linked with IS10A, we did not detect any IS element surrounding the *bla*_OXA-232,_ gene, suggesting different transfer modes for these genes. Specifically, *bla*_OXA-48_ may transfer independently from the plasmid as an independent unit, while *bla*_OXA-232_ is transferred together with the plasmid. Furthermore, we observed differences in the presence of conjugation genes in the *tra* locus between the two plasmids. (Figure S7A). The plasmids showed a broader geographical distribution beyond the current collection: pIncHIIB and pOXA232 in most external samples, with 78% and 23% of genomes showing coverage above 90% for pIncHIIB and, to a lesser degree, pOXA48 in 45% of all external genomes, respectively (Figure S5). The phylogenetic distribution of the genomes carrying the plasmids suggests both the dissemination of lineages harboring the plasmid across countries and signatures of plasmid transmission between distant countries. Besides the ST2096 clones, the plasmids were also present in other lineages in our collection, providing evidence of HGT among the lineages (Fig. 1). The pOXA232 plasmids was common in ST2096 clone but occurred in multiple other lineages, i.e., < 90% coverage was observed in genomes from four STs other than ST2096. The pOXA48 has a broader phylogenetic distribution and occurred in multiple other lineages from 13 STs other than ST2096. The pIncHIIB was detected in only two ST14 and ST866 other than ST2096 strains. This distinguishes the clone from other clones including the sister clone of ST14 and presumably explain the success of the ST2096 clone (see “Discussion”).

### pIncH1-1B pangenome is dynamic, in particular for the resistance gene cassette

The pIncHI1B plasmid is a large ~ 300kbp mosaic plasmid, i.e., a plasmid with genetic cassettes for virulence and antimicrobial resistance, that shows most of the characteristics of the hybrid plasmid previously reported in ST2096 in India and showed 73% percent sequence identity to this plasmid (Figure S7A outer red layer band in pIncHI1B) [61]. The resemblance between this plasmid and the one reported in India further supports the link between India and the current strains, as described above for SNP clusters. The genomes of the pIncHI1B plasmid exhibited substantial size variation across the strains, with a mean size of 300,411 bp (ranging from 151,761 bp to 321,032 bp), which accounted for 56% of the plasmid mean size. This corresponded to an average 336 genes per genomes with the minimum of 177 and maximum of 363 genes. Pangenome analysis of the plasmids further revealed a dynamic composition comprising 215 core genes present in more than 95% of isolates, 140 genes in the shell genome (present in 15% to less than 95% of strains), and 37 genes in the cloud genome (present in less than 15% of strains). Apart from *dfrA12, msrE*, and *sul1*, other resistance genes exhibited variable presence within the cohort of genomes under study. In contrast, the virulence factor cassette, composed of the *iuc* locus and *iutA* gene, remained consistently present in all samples with the plasmid (Figure S7B, Figure S8A). The differences in the dynamics of resistance and virulence cassettes were also evident in the pangenome graph of the plasmid (Figure S7B). Antimicrobial resistance genes were found in the highly connected subnetwork of the pangenome network, tightly linked with various IS elements, indicating different combinations of genes on the contigs (Figure S7B). In contrast, virulence genes were stably co-located across the population. The resistance genes and their linked IS elements constituted the entire set of significant co-evolving genes (*p-*value < 0.01 from Pearson correlation test), with three epistatic interactions between pairs of resistance genes (i.e*., aac4*, *bla*_OXA-1_, and *tetA*) (Figure S8B, Figure S8C). The population structure of the plasmid supported a mosaic structure of gene cassettes coming together on the same plasmid backbone. However, it did not indicate long-range co-evolution of genetic interactions between genes, including interactions between antimicrobial resistance and virulence factor genes.

### In-hospital mortality is linked with the genomic characterization of colonizing STs

**Fig. 5:**
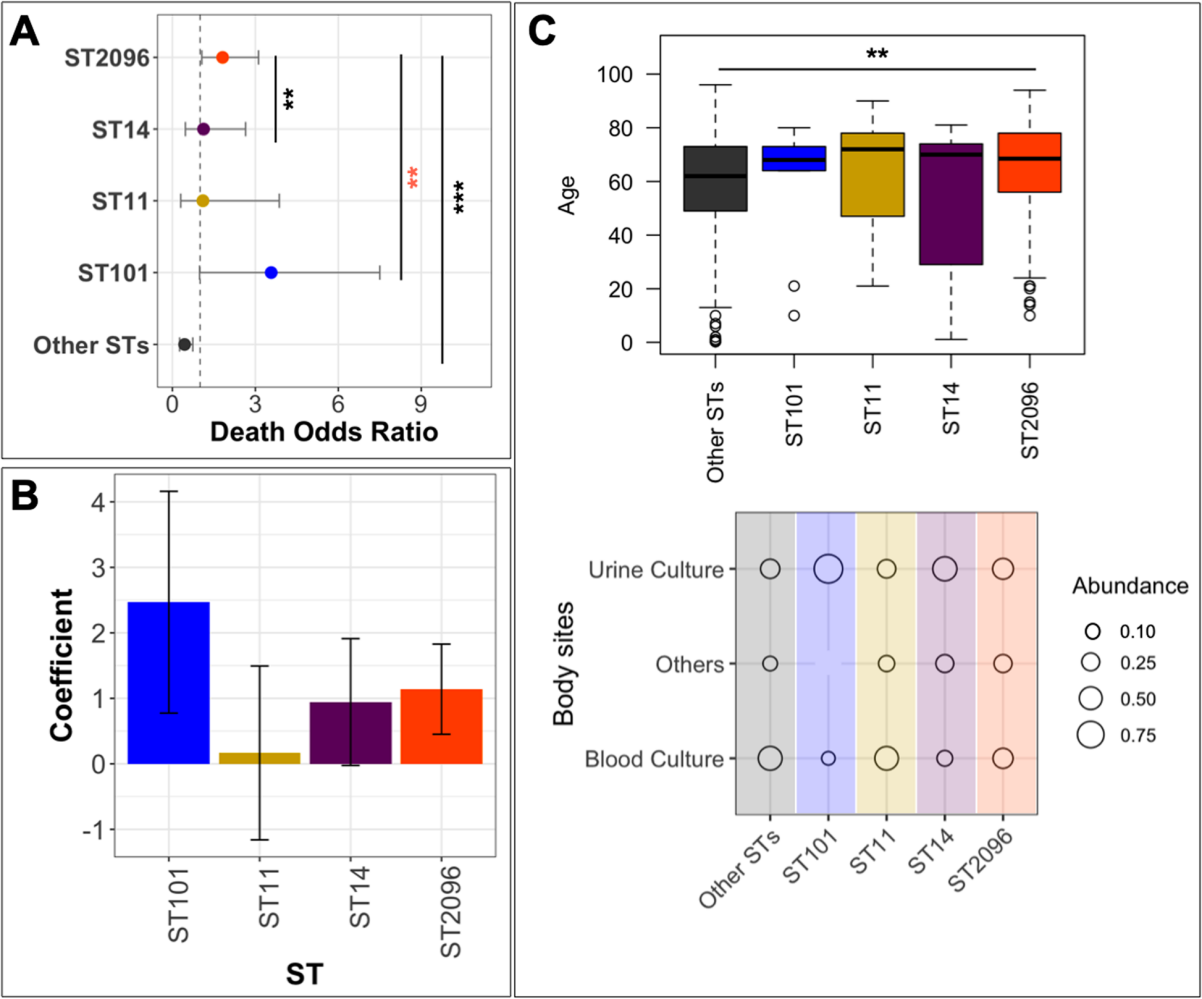
Integration of ST data with clinical and demographic data. **A** The odds ratio of death for the major STs. The error bars show a 95% confidence interval. The dotted line shows an odds ratio of one, above which the presence of STs is positively linked with death. The asterisks ** and *** correspond to *p*-values of < 0.01 and < 0.001 from *Z*-test, respectively. The black and orange colors for the asterisks show the significance when the odds ratio smaller and bigger in ST2096 than the other group, respectively. **B** The coefficient value associated with STs from logistic regression analysis in which sex, gender, age, and site of isolation were included as predictors. The coefficient is the expected change in log odds of being an ST type. **C** Distribution of patients’ age and body site of infection. The ** shows *p*-value < 0.01 from Wilcoxon signed-rank test

**Fig. 6:**
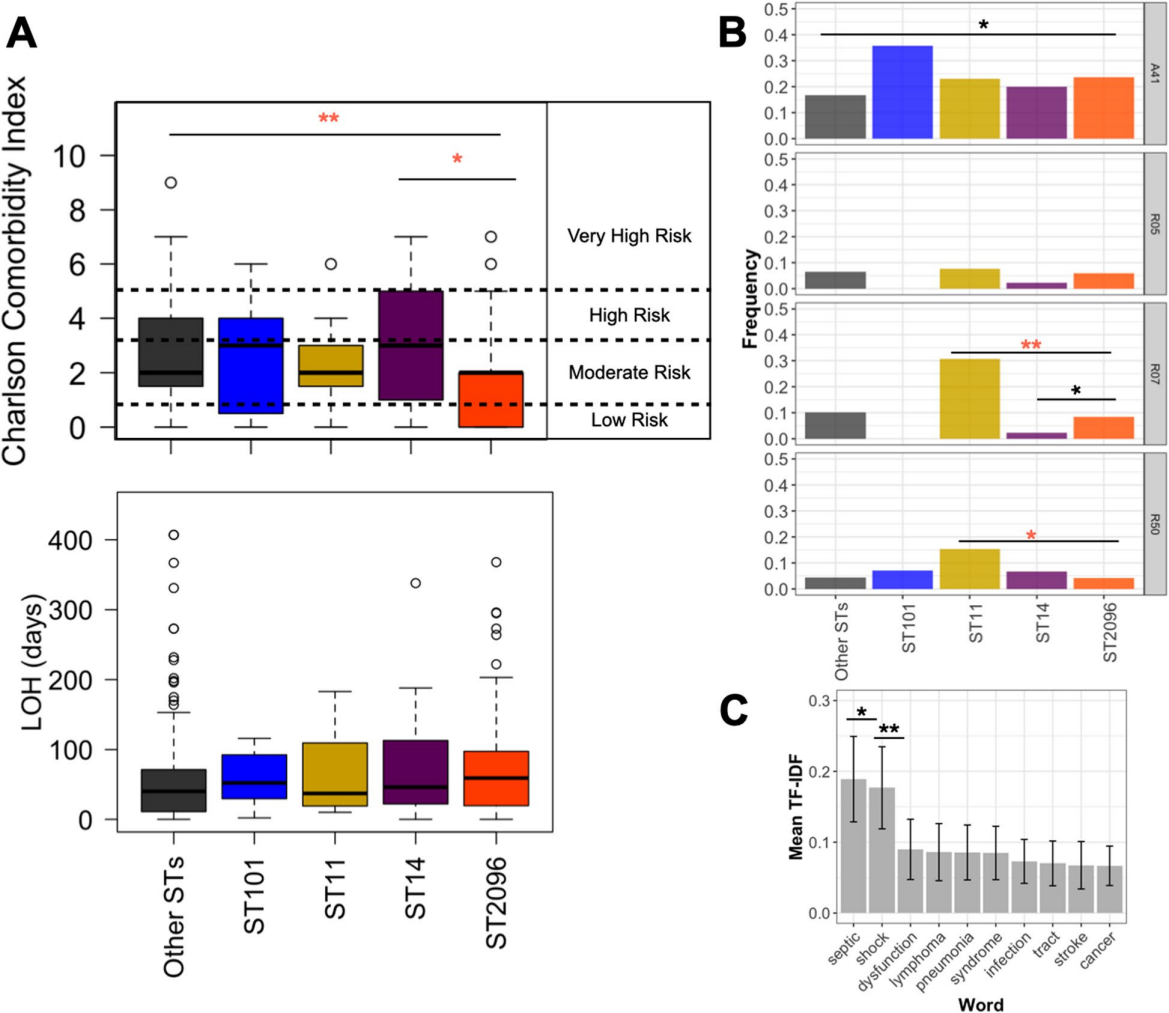
Integration of clinical data for patients with ST data. **A** Charlson comorbidity index and length of hospitalization (LOH) across patients. The index is computed from comorbidities, as detailed in the methods. Length of hospitalization (LOH) in days across in-patients. **B** Relative frequency of patient conditions linked with infections across patient groups carrying different STs, indicated in ICD-10 codes. The codes A41, R05, R07, and R50 correspond to sepsis, cough, pain in throat and chest, and fever, respectively. The horizontal lines in **A**, **B**, and **C** represent the significance between ST2096 and other STs. The statistical test for **A** and **B** was the Wilcoxon signed-rank test, and for **C**, it was the one-sided proportion test. **C** The mean term importance measure (tf-idf) for the topmost important words overrepresented in the diagnostic tests for ST2096. Error bars show 95% confidence interval from 100 bootstrapped samples. The * and ** signs correspond to a significance level of < 0.05 and < 0.01, respectively, based on the Wilcoxon signed-rank test. The black and orange colors for the asterisks correspond to cases where the mean value (in **A** and **B**) and relative frequency (in **C**) for ST2096 are greater and smaller than the other group, respectively

We evaluated the clinical significance of major STs by integrating in-hospital mortality data with the infection data of the dominant STs. The ratio of deceased patients for ST2096, ST11, ST101, and ST14 was 53% (44 out of 83 patients), 45% (5 out of 11 patients), 72% (8 out of 11 patients), and 46% (11 out of 24 patients), respectively. For ST2096 and ST101, these values were found significantly higher than the rate for the rest of the population, i.e., 33% (38/116) (*p*-value from one-sided proportion test < 0.01). The results from the death rate over time indicate that carriage of ST2096 and ST101 is consistently linked to a higher mortality rate compared to the other STs, and this effect becomes significant for ST101 after 30 days and for ST2096 after 90 days (proportion test *p*-value < 0.05) (Figure S9A). The difference is also evident among subpopulations with different virulence gene profiles. The presence of the *ybt* and the concurrent presence of *ybt* and *iuc* loci are linked to in-hospital mortality compared to the single carriage of *iuc* and the lack of both genes, with the difference becoming more significant after 60 days (proportion test *p*-value < 0.05) (Figure S9B). Similarly, the concurrent presence of ESBL and carbapenemase genes turned out to be significantly linked with a higher mortality rate than the single presence of the genes or the absence of the genes after day 60 of hospitalization (Figure S9C). Patients with hypervirulent and multidrug-resistant strains were found to have a slightly higher mortality rate than the rest of the population over time; however, the difference was not significant. This is attributed to the definition of the phenotype, as the single presence of the *ybt* gene, despite the strong link with mortality in our collection, does not classify the strains as hypervirulent (Figure S9D) [27]. In line with this, the odds ratio for death in patients with ST101 and ST2096 was significantly greater than 1 (95% confidence interval of the values), in contrast to ST11 and ST14 and other STs (Fig. 6A). Although for ST2096 the odds ratio is 1 still close to one, the odds ratio was significantly larger than odds ratio for ST14 and the rest of the population (Fig. 5A) (*p*-value one-sided Wilcoxon ranked test < 0.01). For ST101, the infection rate is higher than ST2096 and is closely comparable to a previous report of 72% in infections caused by carbapenem-resistant strains [62]. The significance association of ST2096 and ST11 carriage with in-hospital mortality still held true even after accounting for potential confounding factors such as age, infection site, and comorbidities (Fig. 5B). ST101 is recognized as a dual-risk clone with a combination of hypervirulence and resistance [63], and ST2096 appears to show a similar level of risk (Fig. 5B). ST2096 also displayed demographic and clinical features similar to hvKp, i.e., colonizing individuals of all ages [54, 55], with an age distribution ranging from 11 to 75 (Fig. 5C). Furthermore, ST2096 isolates recovered from different body sites, a characteristic previously reported for hvKp [54, 55]. This contrasts with the ST101 strains in our collection, which was not recovered from urinary tract (UC) colonization/infections and had only 1 out 14 samples (7%) from blood. ST11 strains, exhibited a higher frequency of blood colonization (7/13, 53%, *p*-value from one-sided proportion test < 0.05) than ST2096 strains, although the frequency of strains from urine culture was comparable for ST11 (3/13, 23%) and ST2096 (28/120, 23%) (Fig. 5C). These findings highlight the clinical significance of ST2096, which is underpinned by genetic determinants for virulence and resistance.

### Integration of genomic and clinical data shows links between the ST2096 colonization/infection and sepsis

We evaluated the clinical manifestation of the carriage of ST2096, in comparison to other STs. The results do not reveal a higher Charlson comorbidity index for ST2096 compared to other STs (Fig. 6A). The values are lower for ST2096 and are significantly lower than the rest of the population (Wilcoxon signed-rank test *p*-value < 0.01) and the sister clade ST14 (Wilcoxon signed-rank test *p*-value < 0.05) (Fig. 6A). However, patients with ST2096 strains turned out to have longer hospitalization than those with other STs (Wilcoxon signed-rank test *p*-value < 0.01), suggesting ST2096 carriage is linked with burdens not included in the Charlson comorbidity index (Fig. 6A). We, therefore, evaluated the over-representations of conditions linked with infections. The results show that in ST2096-related notes, the sepsis code < < ICD10 code: A41 > > and chest pain < < ICD10 code: R07 > > are significantly over-represented compared to the other STs and sister ST14, respectively (proportion test *p*-values < 0.05) (Fig. 6B). Patients with ST2096 had more instances of sepsis and chest pain than patients with sister ST14 (proportion test *p*-values < 0.05) (Fig. 6B). No significant difference was observed for the cough symptom < < ICD10 code: R05 > > , and ST2096 representation was smaller than ST101 in patients with fever < < ICD10 code:R50 > > (proportion test *p*-values < 0.01) and chest pain (proportion test *p*-values < 0.01), which is in line with higher mortality for ST101. We further explored the importance of the terms in patients with ST2096. To achieve this, we extracted the most relevant medical terms from the clinical documents for patients with ST2096, comparing them with those noted for patients with other STs. Figure 6C displays these terms and their relevance scores (see “Methods”) for ST2096. Terms related to bacteremia, i.e., < < sepsis > > and < < shock > > , were found consistently as the most important terms after bootstrapped data and were significantly more important than the other terms (Wilcoxon signed-rank test *p*-value < 0.01). For other major STs, the count of terms was too low to yield conclusive results for the comparison of term importance between other STs. Despite this, the findings for ST2096 agree with the higher resistance and virulence levels observed and suggest a potential role of virulence and resistance in the invasive phenotype.

## Discussion

In this study, we conducted a comprehensive and systematic genomic epidemiology analysis of a large-scale *K. pneumoniae* population in a single hospital over 8 years. Our findings revealed a diverse population, with multiple STs responsible for the infections. However, we observed the dominance of strains belonging to CC14, which have been contributing to repeated infections within the hospital setting over time. Specifically, CC14 strains, particularly the ST2096 subpopulation, exhibited a high level of resistance and virulence, as evidenced by the emergence of resistance mutations and genes within a relatively short period. These data underscore the evolution of endemic ST2096 subpopulations among hospitalized patients.

Most studies on hvKp and MDR *K. pneumoniae* have focused on dominant clonal clones, such as ST258, which has a widespread global distribution [64, 65]. However, despite the prevalence of ST258 in Europe and North America, it appears to be rare in other parts of the world [5]. The dynamics of emerging hypervirulent and resistant STs in different regions have not been well-documented. The dynamics of ST258 in the hospital setting have been shown to follow a pattern of clonal expansion, dominance, and eventual extinction over time, with specific lineages persisting [9]. Evolutionary dynamics of ST2096 follow a similar pattern in which subclones of the ST2096 clone with distinctive set of resistance determinants for colistin and carbapenem compete and becomes fixated in hospital setting. Signatures of rapid evolution are also detectable in the plasmid evolution. The in-depth plasmid pan-genome analysis showed a highly mosaic structure of the plasmid with a lack of interaction between virulence and resistance cassettes. This lack of interaction may be attributed to the strong selective pressure on the antimicrobial gene cassette due to the intense influence of antimicrobial treatments or the limited time available for genetic interactions between virulence genes and antimicrobial resistance genes since the recent formation of the mosaic plasmid. The prevalence of the plasmid mosaic appeared to be a discriminating feature ST2096 from the sister clade of ST14, which may account for the success of the clone. However, our ST2096 strains harbored 181 genes that were completely absent in ST14 clade. Future functional genomics analysis may help to fully characterize the potential contribution of these genes to the enhanced pathogenicity in ST2096, as compared with the ST14 clade.

The evolution of ST2096 may also take place beyond hospitals. A recent genomic survey of a smaller collection of ST2096 in India demonstrated the prevalence of these strains not only in clinical settings but also in the community [24]. In line with this, we detected three strains with confirmed community-acquired infections. These findings suggest that ST2096 is a circulating clone in the community that has been introduced into hospital settings on multiple occasions and undergoes rapid genome evolution. The broader regional expansion of the ST2096 clone in the Middle east aligns with Saudi Arabia serving as a touristic and religious hub for the regional countries, potentially acting as a melting pot for transmitting multi-drug resistant strains [66]. For a more comprehensive understanding, a more extensive collection from the community would help us ascertain whether the emergence of novel resistance occurs in hospitals or the community.

In this study, we have successfully integrated clinical data with genomic data to quantify the risk associated with the length of hospital stay. Understanding the impact of antibiotic resistance in Gram-negative nosocomial infections is crucial, and one key factor is the increased length of hospital stays. Previous studies have indicated longer stays for patients infected by multidrug-resistant (MDR) strains, showing notable differences between those colonized by MDR and susceptible strains [67, 68]. Building upon these findings, our research extends the understanding by revealing a direct link between the duration of hospitalization and a patient’s importance in the transmission network within the hospital. Our results strongly suggest that persistent carriage of these strains could lead to higher transmission rates, thereby contributing to prolonged hospital stays. Although the precise nature of increased indirect *K. pneumoniae* transmissions remains unclear and could stem from various sources, such as colonization [69], equipment, or hospital staff [70, 71], our findings underscore the necessity for tailored treatment plans for patients with extended hospital stays. This could involve interventions to decolonize patients or further characterization of the colonizing strains to identify potential strategies for containment and prevention. By addressing these factors, we can make significant strides in curbing the impact of antibiotic resistance and improving patient outcomes in nosocomial infections.

Despite the depth and breadth of the collection analyzed here, this study has several limitations. Firstly, since the collection was retrospective and based on routine hospital screenings, there might be potential biases in sampling and changes in the clinical importance of samples, especially those from specific sites like blood cultures. This has led to a disproportionate representation of strains in the collection, limiting the overall understanding of infection dynamics from different sites. Secondly, the collection is highly underrepresented in samples from the community, making it challenging to assess the contribution of hospital-acquired versus community-acquired routes of infections. Finally, despite the detailed metadata available, the electronic health record (EHR) for some patients was incomplete and did not include information about the progression of the infection, details of the treatment, and patient movements between wards during the hospitalization period. This limitation of the completeness of the EHR data has been recognized for research in the infectious diseases [72], as the data is primarily produced for clinical management and not for systemic research analysis [73]. A prospective controlled study with improved metadata will be necessary to address these limitations and obtain a higher-resolution map of transmissions in the hospital. Such a study will also help to establish links between within-patient and between-patient dynamics.

## Conclusions

*K. pneumoniae* infections are recognized as a pressing health concern, yet the emerging CP clones lack adequate characterization. In this study, we aimed to shed light on the emerging hypervirulent ST2096 clones and highlight their clinical significance. By integrating genomic and clinical data, future research can discern the differences in pathogenicity mechanisms between the emerging STs, such as ST2096, and other globally dominant hypervirulent strains, such as ST258. This, in turn, will aid in determining the relative risk level of these strains in clinical settings. Given that a single outbreak of hvKp strains could result in their widespread dissemination, conducting such comparative analyses will help us better understand the importance of monitoring infections caused by these emerging hvKp clones to prevent outbreaks in healthcare settings. Moreover, this information will facilitate the identification of specific genes responsible for the biological success and virulence of these emerging clones, providing valuable insights for designing targeted drugs.

### Supplementary Information

**Supplementary Material 1. **

**Supplementary Material 2. **

## Data Availability

Genomic data for the short-read and long-read sequencing were deposited in the European Nucleotide Archive (ENA) under the study accession PRJEB36683 (https://www.ebi.ac.uk/ena/browser/view/PRJEB36683). The processed data has been uploaded to the GitHub directory of the paper (https://github.com/DaneshMoradigaravand/KlebsiellaPneumoniaeMNGHA). The corresponding metadata for the genomes are detailed in Supplemental Table S1.
