# Supplementary material for: The emergence of highly resistant and hypervirulent Klebsiella pneumoniae CC14 clone in a tertiary hospital over 8 years
Source: Genome Med. 2024 Apr 18;16:58. doi: 10.1186/s13073-024-01332-5 (PMC11025284; doi:10.1186/s13073-024-01332-5)
Supplement: Supplementary file 2 — Supplementary Material 2. [file 13073_2024_1332_MOESM2_ESM.pdf]

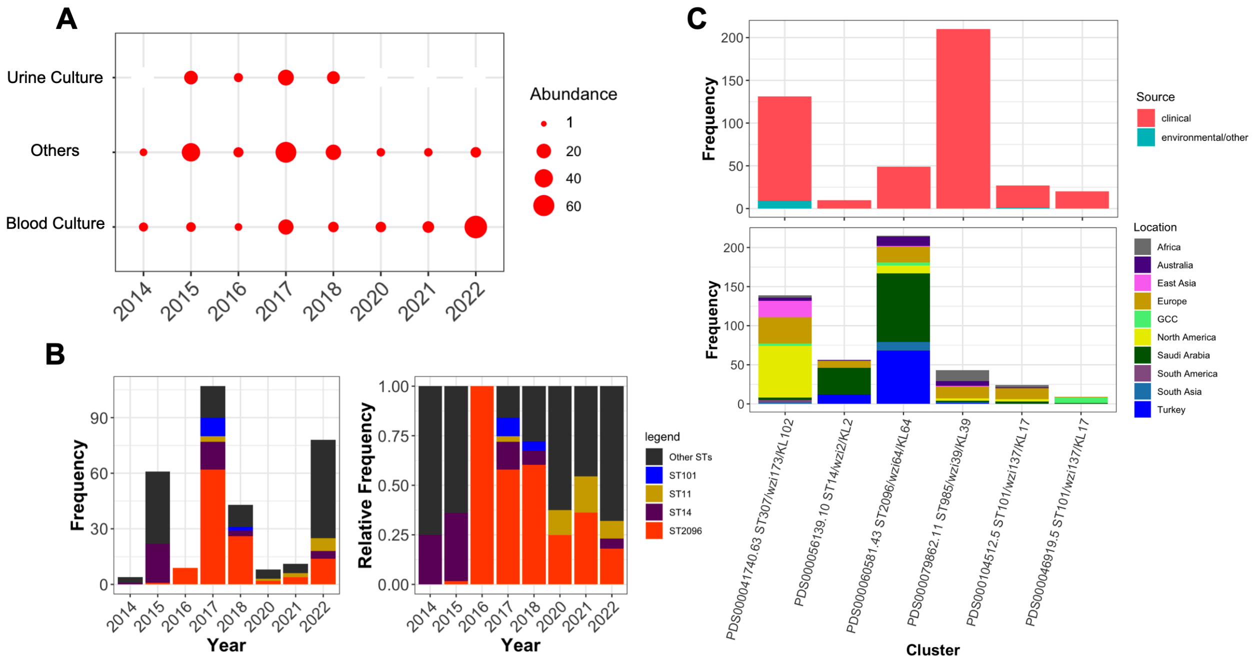

Fig S1 Frequency of source and STs in the collection. A) The distribution of sources of isolates over eight years. B) Absolute and relative frequency of STs over eight years. Other STs have STs with fewer than ten representative samples in the collection. The relative frequency corresponds to the count of STs. In a year, divided by the total number of isolates from the same year. C) The distribution of strains that fell under the same SNP cluster, as defined by Pathogen Detection, as one sample in our collection. Distributions for the location of isolation and sample types are illustrated.

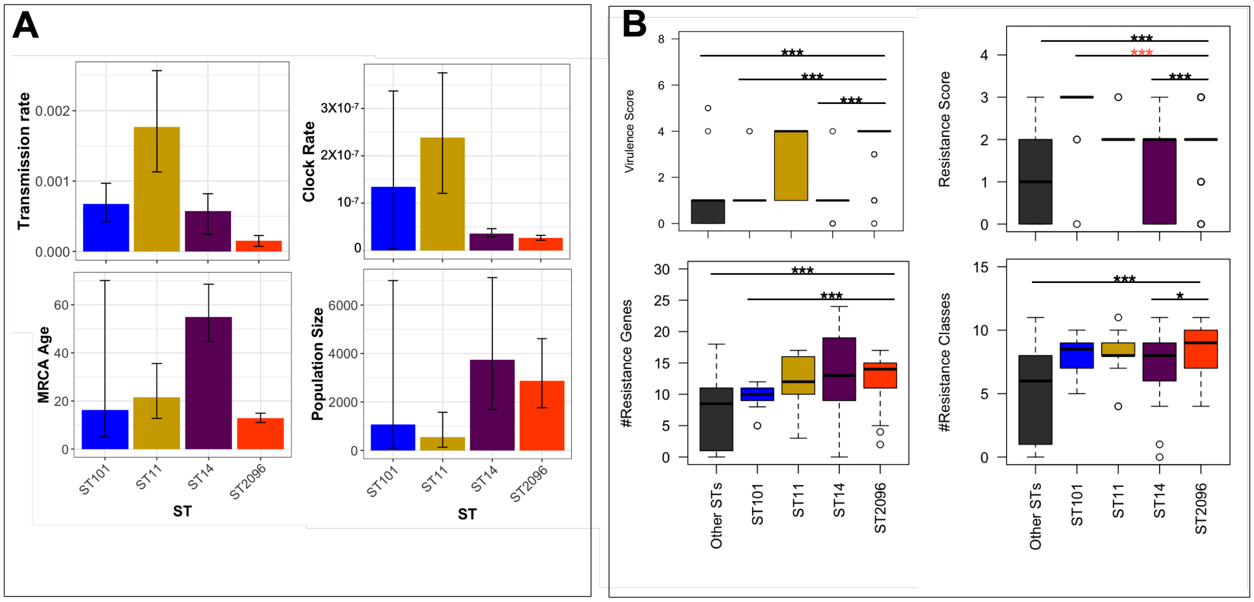

Fig S2 Comparative analysis of STs resistance, virulence and dynamics. A) Epidemiological features from phylodynamic analysis by SCOTTI for STs. The units for MRCA age, transmission rate and clock rate are years, transmission per individual per year and substitution per site per year, respectively. The error bars correspond to 95% credible interval. B) resistance and virulence score and resistance genes/classes count for the major STs and minor STs combined. The boxes show the distribution of the feature in the isolates belonging to the ST group, with dots showing the outliers. For a definition of virulence and resistance score see Methods. The * and ** sign correspond to <0.05 and <0.01 significance level from the Wilcoxon signed-rank test, respectively. The orange and black colors for the asterisks correspond to cases where the mean value (in A and B) and relative frequency (in C) for ST2096 are greater and smaller than the other group, respectively.

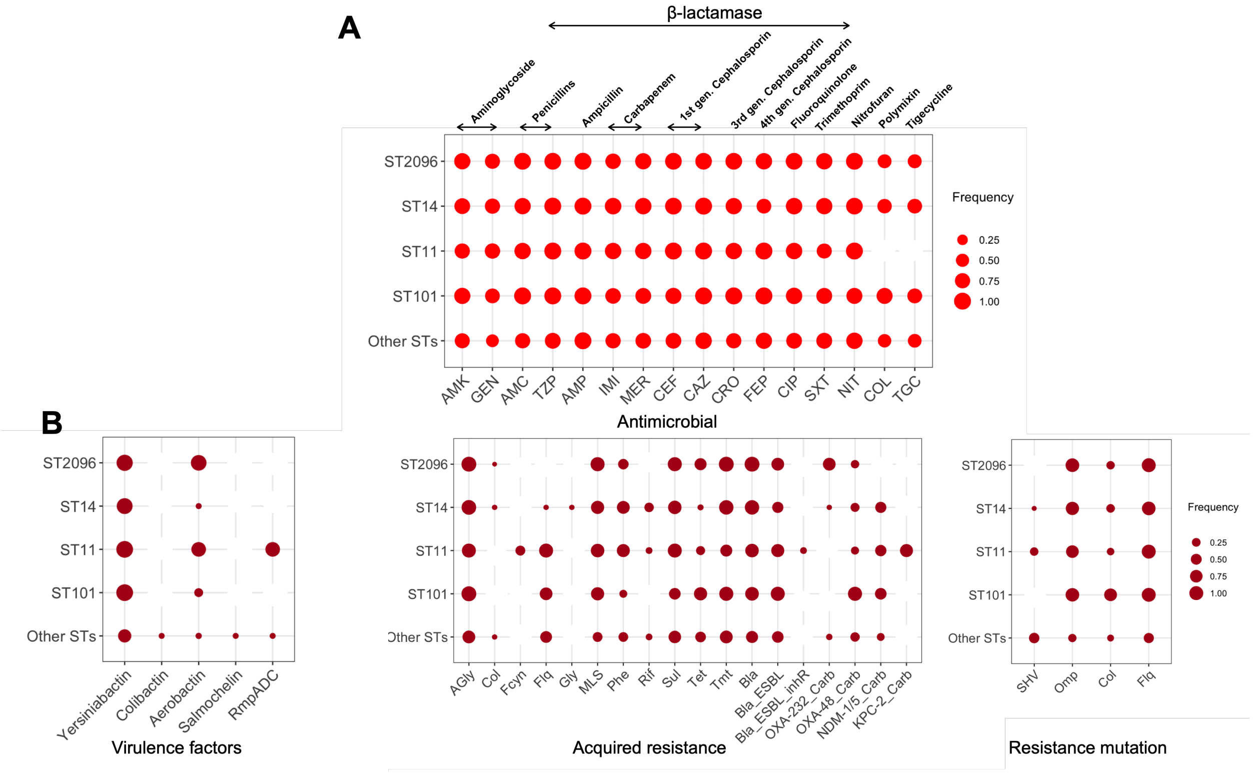

Fig S3 Distribution of antimicrobial resistance phenotype and genotype and virulence genotypes for major clones. A) Relative frequency of resistance phenotypes against different drugs. B) Relative frequency of virulence factor genes, acquired antimicrobial resistance genes, and mutations. Abbreviations in genes are detailed in the caption of Figure 1.

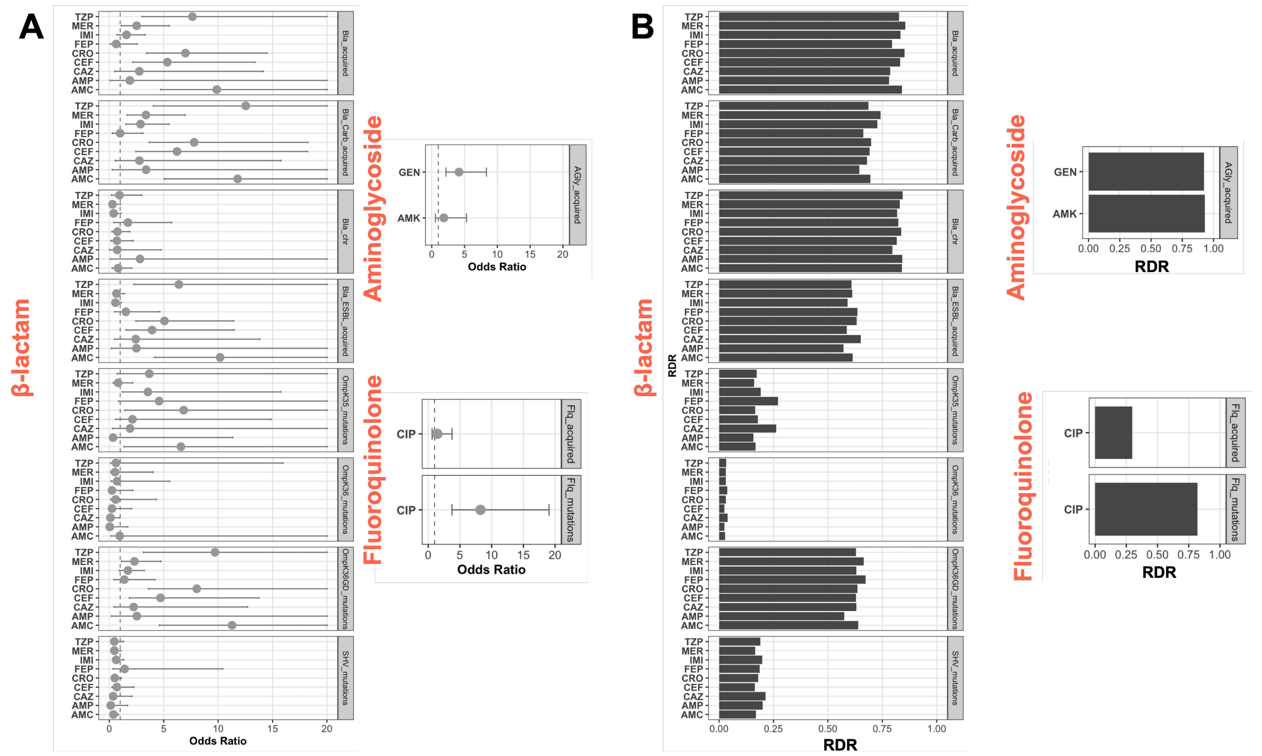

Fig S4 Integration of phenotypic resistance data and known resistance determinants. A) Odds ratio of resistance for different resistance determinants in three classes of antimicrobials. The error bars correspond to a 95% confidence interval. B) Resistance detection rate (RDR) for different resistance determinants in three classes of antimicrobials. The rate corresponds to the number of resistant strains that carry the determinant divided by the total number of resistant strains for each antimicrobial. Abbreviations in genes are detailed in the caption of Figure 1.

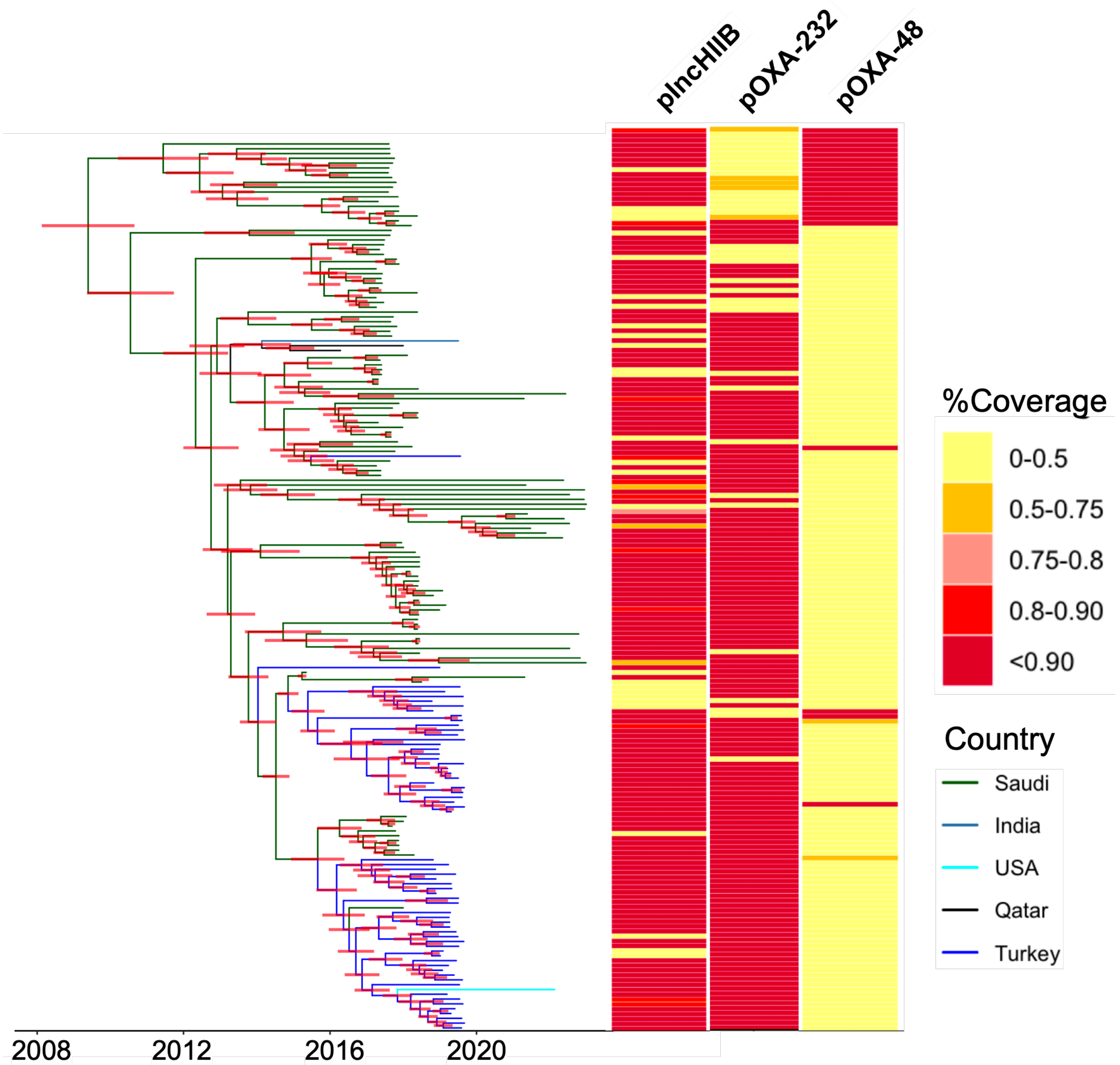

Fig S5: phylogeographic analysis for ST2096 strains in Saudi Arabia and globally. Only strains with an available date of collection were included. The tree is a dated Bayesian tree resulting from 1961 SNP sites for 185 samples in total. The horizontal red bars on the tree show the 95% Highest Posterior Density (HPD) for the age of the node. The colour of ancestral branches represents the most likely location (country) of the ancestors. The heatmap strips display the percentage coverage of plasmids recovered from long-read sequencing data for the entire collection.

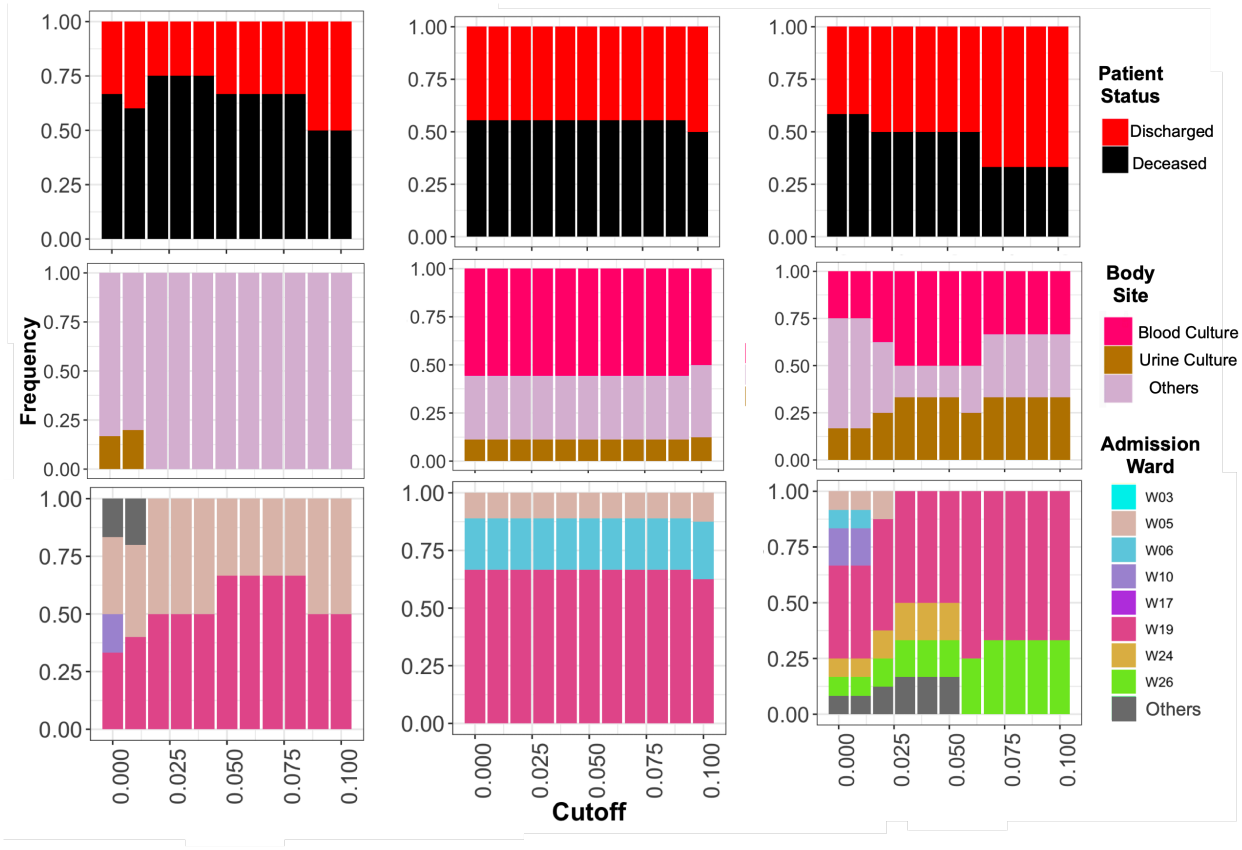

Fig S6 Transmission dynamics of other major STs. The distribution of patient status, body site, and admission hospital ward in networks with different cut-offs for the transmission routes for major STs other than ST2096, i.e., ST14, ST11 and ST101.

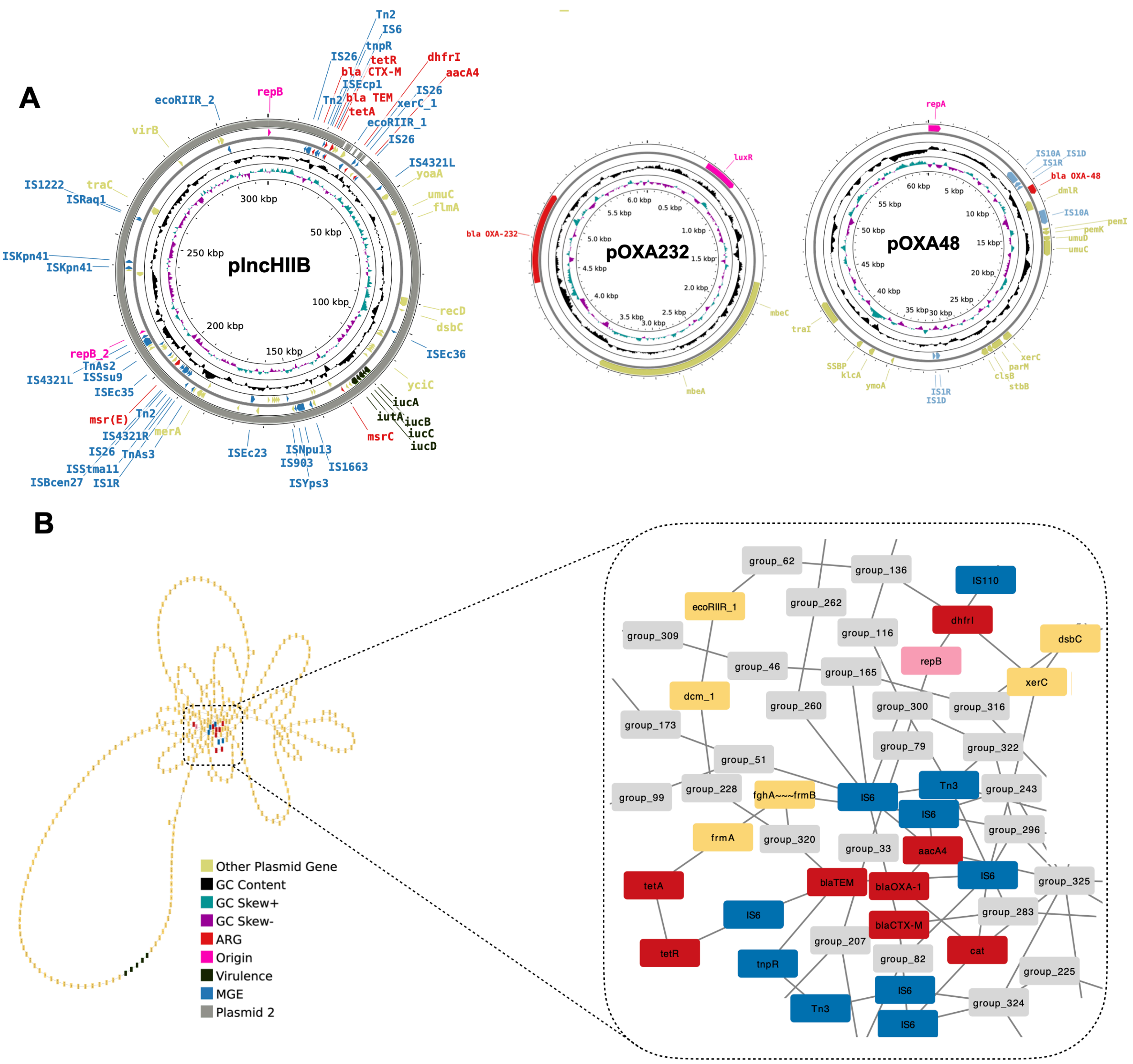

Fig S7 Plasmid structures and pangenome A) Genomic map of ESBL (pIncHIIB) and Carbapenemase plasmids in the ST2096 clones. For each plasmid, we selected the longest plasmid contig in the collection. For the H1-1B plasmid, we did not show the mobile genetic elements to improve presentation. Detailed information on the plasmids and genes is provided in the Supplementary Material. B)Pangenome graph for the pIncHIIB plasmid. Each node corresponds to a gene family. Edges denote connections between the two nodes if the nodes are neighbors on a contig in any sample from the population. The annotation of the genes is provided in the GitHub directory for the project.

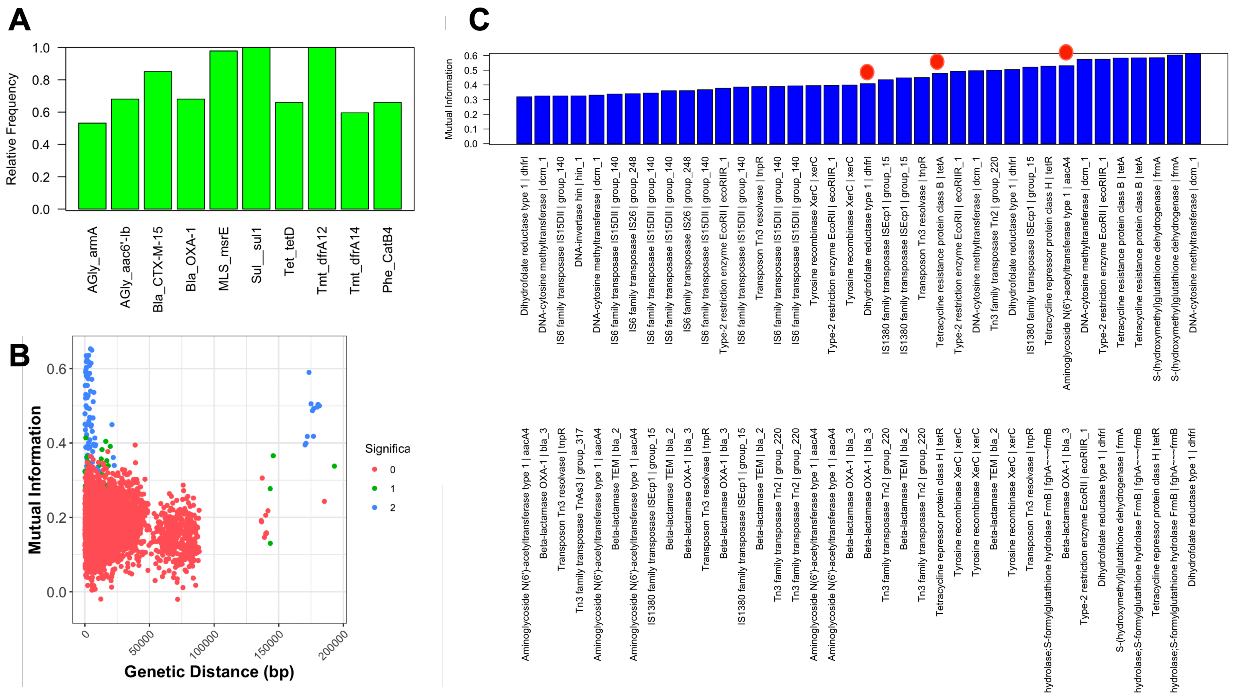

Fig S8 A) Frequency of genes for antimicrobial resistance in the pan-genome for the pIncHIIB plasmid in the ST2096 population. B) Epistatic interaction analysis from the pan-genome data for the pIncHIIB plasmid. The mutual information versus genetic distance for the pairs of genes in the pangenome. Colors correspond to the significance level of mutual information, with 2 and 0 corresponding to the highest and lowest significance, respectively. C) gene pairs with significant values for the mutual information, i.e., epistatic interaction score. The circles denote a pair of known resistance genes.

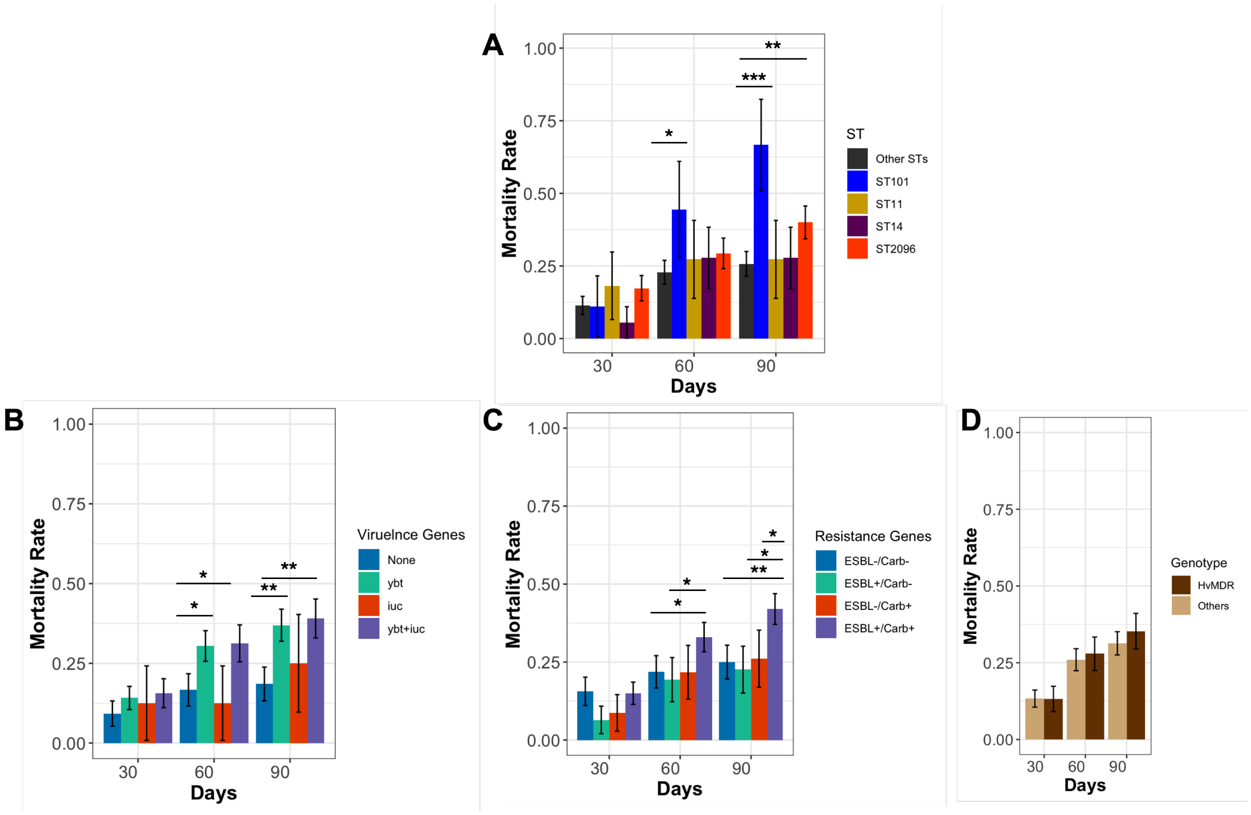

Fig S9 The overall mortality rate identified in the collection for patients carrying different A) STs, B) key virulence factor genes, C) key beta-lactamase gene profiles and D) hypervirulent-resistant as defined in Figure 3. The mortality rate is the reported in-hospital mortality rate and does not include the deaths occurred after the patient's discharge. The stars correspond the significance of the one-sides proportion test, with *, ** and *** corresponding to significance level 0.05, 0.01 and 0.001 respectively. The hospital bars show the bars compared.
